# Efficient and Accurate Ligand Strain Calculations in Solution with the AIMNet2 Neural Network Potential

**DOI:** 10.1021/acs.jcim.6c02468

**Published:** 2026-10-21

**Authors:** Hatice Gokcan, Olexandr Isayev

**Affiliations:** † Department of Chemistry, Mellon College of Science, 6612 Carnegie Mellon University, Pittsburgh, Pennsylvania 15213, United States; ‡ Department of Materials Science and Engineering, 6612 Carnegie Mellon University, Pittsburgh, Pennsylvania 15213, United States

## Abstract

Conformational strain, the energy a ligand pays to adopt its bound conformation, contributes to binding affinity but is routinely misestimated. The standard estimate references a single gas-phase energy minimum. This ignores solvent and mishandles charged ligands: in vacuo their intramolecular electrostatics collapse them into compact minima that deflate the unbound reference and inflate the apparent strain. We introduce AIMNet2-CPCM, a neural network potential trained to B97-3c energies and forces in a conductor-like continuum solvent (CPCM). It treats neutral and charged species on the same footing, and its batched GPU inference makes a solvated global-minimum search practical across an entire screening library. Across ∼8K PDB ligands the solvated strain has a median of 4.0 kcal/mol and rises with net charge. The trend is well determined through a net charge of −2. The −3 and −4 polyanions dominate the high-strain tail, where continuum solvation is least reliable, and their values are reported as upper bounds. Gas-phase force fields reproduce the neutral strains but overstate the charged ones, because they collapse the unbound minimum that solvent screening keeps open. Adding a continuum solvent to the force field removes most of this error and confirms the mechanism. Profiling at this scale further shows that the same ligand strains very differently across the targets that bind it: ligand strain is a property of the bound pose, not a transferable molecular descriptor.

## Graphical abstract

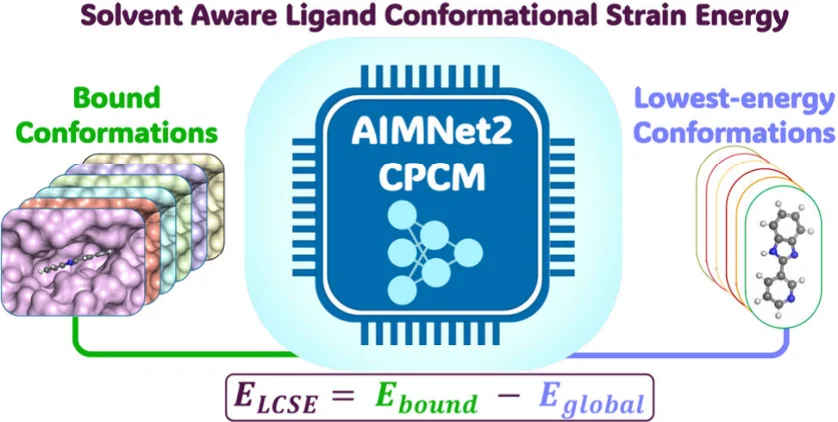

## Introduction

Binding affinity has several components. One is the energy a ligand requires to adopt its bound conformation, the conformational reorganization penalty commonly called ligand conformational strain energy (LCSE). This term matters both for assessing poses in structure-based virtual screening and for improving affinity by preorganizing (rigidifying) a compound toward its bioactive conformation during lead optimization.^1–9^ Rigidification acts on two terms at once. It lowers the enthalpic penalty of adopting the bound conformation, and it reduces the conformational entropy lost when a flexible ligand is immobilized in the pocket.^9^ LCSE as computed here is an energy difference between two conformers and captures only the first term. It is a component of the binding free energy, not a proxy for it, and the entropic contribution can be of comparable size.^9,19^ Strain is nevertheless estimated poorly in routine practice. The usual reference is a single gas-phase minimum, which ignores solvent and mishandles charged ligands whose electrostatics collapse in vacuo.^4,10,19,21^

LCSE is typically computed by subtracting the energy of the ligand’s unbound-state conformation from that of its bound-state conformation, both after energy minimization.^10–13^ While the computation itself is straightforward, the scheme varies considerably. The schemes differ first in how the unbound state is defined. One approach is to perform a conformational search to identify the global minimum as the unbound-state conformer.^13–15^ This approach is faithful to the definition of LCSE, but there is no consensus on the sampling strategy that identifies the global minimum reliably.^13,15,16^ Established alternatives to systematic or stochastic torsion sampling include low-mode search, which follows soft vibrational modes to escape local minima,^81^ and Tork, which drives torsional moves along low-frequency modes of the torsion-space Hessian.^82^ The QM-VM2 mining-minima algorithm goes further and applies QM energy corrections inside the conformational search rather than after it.^83,84^ This addresses the case where the sampling force field and the scoring QM method disagree on which conformers are lowest in energy. The same disagreement appears in our comparison against a force-field-sampled reference set (Figure 2c). The absence of a consensus is especially disadvantageous when the compounds of interest are flexible molecules or macrocycles, for which the global minimum might be very close in energy to several other conformers. In such cases the strain is effectively lower because the ligand has pre-existing conformers close to the bound shape in energy, so the sampling strategy determines the result. Another approach, referred to as local ligand strain energy, seeks to find the minimum energy conformation closest to the bound-state conformer of the ligand.^11,17,18^ While this approach is computationally less expensive relative to the global-minimum search, it fundamentally underestimates the LCSE.^19^

Studies of LCSE also differ in the level of theory used for the energies. One of the main reasons for this difference is the computational cost associated with the conformational search, which is a bottleneck for high throughput drug discovery. The most cost-efficient approach in LCSE calculations is the use of classical force fields (FF) to locate unbound-state conformation which can also be refined with quantum mechanical (QM) or hybrid quantum mechanical/molecular mechanical (QM/MM) calculations.^10,12,13,19–21^ However, the use of classical FFs is limited by well-documented errors in torsional profiles.^22–24^ These errors can misplace the minimum and make LCSE unreliable. A less cost-efficient approach is to employ QM or QM/MM calculations for the computation of LCSE.^11–13,17,25–28^ These approaches, even if they can provide reliable results, can only be suitable for studies handling small-scale libraries due to the high calculation cost. This cost can be reduced with faster approximate QM methods like semiempirical approaches. An example of such cases is the use of open-source Conformer–Rotamer Ensemble Sampling Tool (CREST)^29,30^ which employs extended tight-binding level (xTB) calculations based on GFNn-xTB methods^31^ as well as implicit solvation models. This tool performs well in a range of applications,^32–35^ but it inherently relies on the accuracy of the semiempirical method.

Machine-learned interatomic potentials (MLIPs) combine near-QM accuracy with force-field cost and can drive conformational searches.^36–45^ Conformational energies of neutral ligands have been computed with MLIPs such as ANI and MACE.^15,16,18^ However, current MLIPs are trained on gas-phase energies and forces and do not account for solvation. A ligand is always screened, by the binding pocket when bound and by water when free. The unbound reference is therefore the lowest-energy conformer in aqueous solution, not in vacuum. A gas-phase calculation ignores this screening and, for charged compounds, produces unphysically strong intramolecular electrostatics and collapsed geometries.^10,13,20,21,46,47^ Gas-phase MLIPs therefore inherit the same limitation as gas-phase force fields for LCSE.

We compute ligand strain in solution at screening scale. The energetics come from AIMNet2-CPCM, an MLIP trained to B97-3c energies and forces in a conductor-like continuum (CPCM). It treats neutral and charged species consistently and reproduces solvation effects that gas-phase potentials cannot. For each ligand we search the conformational space in solution and locate the global minimum. This search is practical because the potential optimizes millions of conformers on GPUs, 8.2 million in this work.^38,44,48,49^ The solvation correction is largest, and least ambiguous, for the unbound reference. In vacuo, polar and charged ligands relax into collapsed global minima held together by intramolecular hydrogen bonds or salt bridges. These minima are artifacts of unscreened electrostatics: they deflate the reference energy and inflate the apparent strain. Searching for the global minimum in continuum solvent removes the artifact.

Our central result is that standard gas-phase, single-conformer estimators misestimate the strain of charged drug-like ligands, and that a charge- and solvent-aware potential removes this error at screening scale. Three further results follow. Solvated strain rises with net charge. The trend is well determined through charge −2, and the values for the −3 and −4 polyanion tail are reported as upper bounds because continuum solvation is least reliable there. The strain is insensitive to crystal-structure quality. It also accumulates across many torsional changes rather than one large rotation. Because the method runs at screening scale, we then profile strain across enzyme classes and across ligands that bind more than one target, the two analyses that prior works have not addressed at this scale. The multitarget comparison shows that strain is target-dependent rather than a fixed molecular property: the same ligand can strain very differently depending on the pocket that binds it.

## Methodology

Ligand conformational strain energy (LCSE) calculations were performed using the LigBoundConf data set introduced by Tong and Zhao.^13^ The original data set contains 7887 compounds at their bound state. The subsets and the analyses performed with them are summarized in Table 1. The *Zhao-Set* is a subset of LigBoundConf and consists of bound-state and global-minimum conformations of 6672 compounds for which LCSE was successfully computed with QM calculations.^13^ The *Zhao-Set* is used to conduct benchmark calculations to assess the new AIMNet2 model trained to CPCM reference data. The second subset, *EC-Set*, consists of compounds which are not annotated as cofactors and with a target that has an identified enzyme class (EC) according to PDBe data. The number of ligands decreases across successive steps for several reasons. Ligands were removed when they contained elements outside the 14 supported by AIMNet2, when conformer generation or geometry optimization failed, and, for the annotation-based subsets, when the cofactor or enzyme-class metadata was not available from PDBe. The Zhao-Set thus contains the compounds for which reference bound and global-minimum conformations were available from Tong and Zhao, and the EC-Set contains the noncofactor ligands for which a target enzyme class could be assigned.

**Table 1:** Dataset and Subsets, Their Compositions and the Analysis They Are Used for in This Work

data set	composition	analysis type
LigBoundConf^13^	∼8K ligands obtained from PDB	Conformational energies with new AIMNet2 solvation model
Zhao-Set	A subset of LigBoundConf^13^ data set with successful 6672 LCSE calculations; It contains bound-state and global minima conformations	The distribution of the LCSE with respect to the molecular properties and distribution of binders with multiple targets
EC-Set	Subset of LigBoundConf^13^ with ligands that are not annotated as cofactors and with a target that has identified enzyme class according to PDBe	The distribution of LCSE with respect to target EC

### AIMNet2-CPCM Model

The AIMNet2-CPCM model is trained on the same reference data points as the gas-phase models (∼20M) but with reference energies and forces computed with the B97-3c composite method^48^ in the conductor-like polarizable continuum model (CPCM)^49^ with water () as the solvent. B97-3c combines a reparametrized B97 GGA functional with the def2-mTZVP basis set, a modified def2-TZVP with reduced polarization. It adds the D3 dispersion correction with Becke–Johnson damping^51,52^ and a short-range bond (SRB) correction for basis-set incompleteness. All reference calculations were run with ORCA 5.0.3.^50^ B97-3c was chosen as a pragmatic reference. It is a low-cost composite method that makes reference energies and forces for the full training set of ∼20 M points tractable, and with CPCM it provides a consistent solvated reference for neutral and charged species. Reported LCSE values inherit the accuracy limits of this reference level (see Limitations), and the QM accuracy of AIMNet2-CPCM should be read as accuracy relative to B97-3c/CPCM rather than to a higher-level benchmark. The AIMNet2 training data sample neutral and charged organic chemical space broadly and were not curated from the LigBoundConf ligands. The benchmark on the Zhao-Set is therefore a test of transferability to drug-like ligands, not an evaluation on training examples.

As in the gas-phase models,^44^ AIMNet2-CPCM predicts energies and forces for neutral and charged molecules composed of 14 elements (H, B, C, N, O, F, Si, P, S, Cl, As, Se, Br, I). It combines physics-based long-range and ML-parametrized short-range terms. A schematic overview of AIMNet2 architecture can be seen in Figure 1. AIMNet2 separately computes local configurational interaction energy in implicit solvent (), and dispersion correction (), to compute the total energy of a given system. Partial charges are inferred from the feature vectors and refined iteratively as part of the message-passing procedure while Neural Charge Equilibration^38^ (NQE) is applied after each partial charge update.

**Figure 1:**
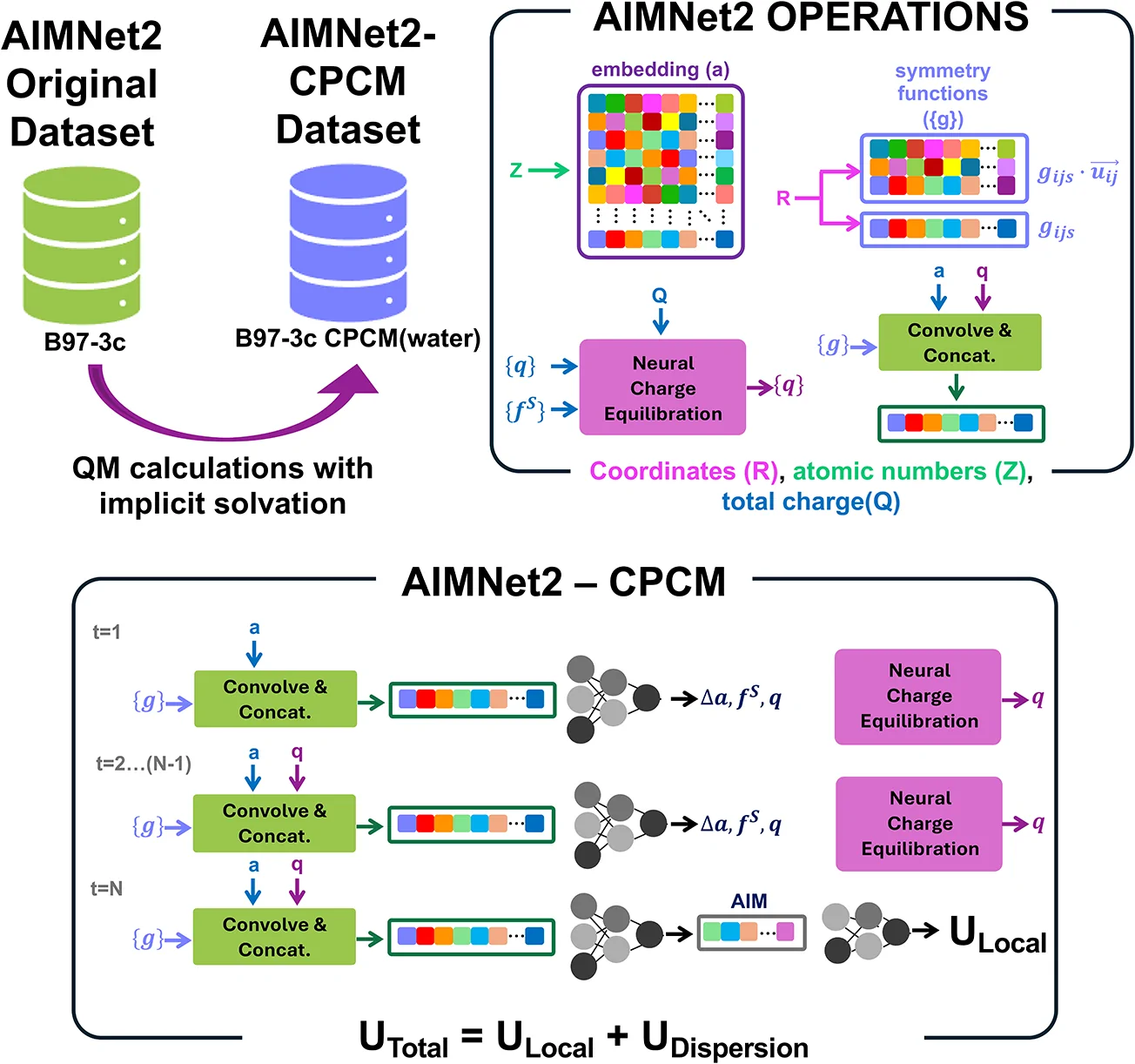
Architecture of the AIMNet2 which uses a message passing approach. The inputs of the model are atomic coordinates (), atomic numbers (), and net charge of the system (). Atomic feature vectors () are calculated via convolution and concatenation of atomic embeddings () and atom-centered point charges () while net charge is maintained with neural charge equilibration (NQE). The total energy () is calculated by the summation of dispersion () contribution with the local energy in implicit solvent () that is obtained by using atoms-in-molecule vector (.

### Bound-State Energy

The bound-state energies were obtained after optimizing the geometries of bound-state conformations while applying torsional constraints using Atomic Simulation Environment^53^ (ASE). Torsions were frozen while bond lengths and angles were relaxed. The bound-state energy therefore reflects the experimentally observed torsions, which encode the strain imposed by the pocket, without the bond and angle distortions of crystallographic refinement that would otherwise dominate the energy. Because the frozen torsions are taken directly from crystallographic coordinates, refinement and coordinate error propagate into the bound-state energy and therefore into the per-ligand LCSE. Individual LCSE values therefore carry an uncertainty, larger for lower-resolution structures, and are best interpreted in aggregate rather than as exact single-ligand penalties.

### Global-Minimum Search

To locate global-minimum conformations, we generated up to 5000 conformers per ligand with the Omega Toolkit (OpenEye).^54^ Omega was run with its default search force field, MMFF94s-mod without Coulomb terms (mmff94smod_NoEstat). The optional Sheffield continuum solvation term was not used. Two Omega defaults were changed. The maximum number of conformers was raised from 200 to 5000, and the energy window for conformer retention was raised from 10 to 999 kcal/mol, so that no conformer was discarded on the basis of its force-field energy. The force field therefore served only to generate geometries, and every energetic decision was made on the AIMNet2-CPCM surface. Because the search force field omits electrostatics, the generator cannot collapse charged conformers, and the solvated and gas-phase minima are both reachable from the same pool. To avoid duplicate conformations, we applied an RMSD threshold that increases with the number of rotatable bonds, from 0.2 Å to 0.8 Å in steps of 0.2 Å per three additional rotatable bonds, with hydrogen sampling enabled. Across the 7887 ligands this produced 8.16 million conformers, a median of 644 and a mean of 1035 per ligand (Figure S14). Only 223 ligands (2.8%) reached the 5000-conformer cap. These are concentrated among ligands with 3 to 5 rotatable bonds rather than the most flexible ones, because the RMSD threshold rises with rotor count. Every conformer was geometry-optimized with AIMNet2-CPCM using an in-house batched optimizer on GPUs, and the lowest-energy optimized conformer was taken as the global minimum.

### Computational Cost

The global-minimum search optimized 8.16 million conformers for 7887 ligands, a mean of 1035 per ligand. The production runs used one member of the AIMNet2-CPCM ensemble with an in-house batched optimizer on NVIDIA V100 GPUs (Expanse, SDSC; Delta, NCSA). Those runs were not timed at the conformer level, so we remeasured the cost on one NVIDIA L40S with the same single-member model and a production-grade batched optimizer. The test set was 1024 RDKit conformers of 64 ligands drawn from the library, and CPU reference timings for the alternatives were measured on the same hardware (Table S3). Two regimes must be distinguished. The first is a single CPU core, which is not the platform AIMNet2 is designed for, because the PyTorch implementation is not optimized for CPU execution. There, one unbatched geometry optimization of a drug-like ligand takes a median of 3.0 s with the AIMNet2-CPCM model, 7.7 s with GFN2-xTB/ALPB and 0.05 s with MMFF94. The second regime is batched inference on the GPU. The single-member forward pass processes 10 500 conformer-steps per second at a batch size of 256, about 240 times the rate of one CPU core. The complete batched optimization sustains 22.7 conformers per second at a batch size of 512, with 1022 of 1024 conformers converged in a median of 161 steps. After optimizer overhead and the stricter convergence criteria, this is 70 times the per-conformer CPU throughput. Unbatched GPU execution, one conformer at a time, is launch-bound. With the full four-member ensemble, one optimization takes 3.7 s on the L40S against 11.6 s on one CPU core, a factor of 3 rather than 240 (Table S6). Batching, not the GPU alone, is what makes the method fast. At the measured batched rate the 8.16 million conformers of this work correspond to about 100 L40S-hours, or 46 GPU-seconds per ligand. The same search with GFN2-xTB/ALPB would need on the order of 17 000 CPU-core-hours. A metadynamics-based CREST search at the GFN2-xTB level is the semiempirical alternative for locating solvated global minima. It has been reported to be about 400 times slower than a 20-conformer NNP protocol that itself averages 44 s per ligand on a GPU,^16^ which extrapolates to roughly 5 CPU-hours per ligand and 4 × 10^4^ CPU-hours for this library. MMFF94 remains the cheapest per conformer by 2 orders of magnitude on either platform, but Figures S15 and S16 show that its strain is unreliable for charged ligands. The cost of AIMNet2-CPCM does not depend on net charge and scales near-linearly with system size,^44^ which makes a solvated, charge-aware global-minimum search practical across a screening library.

### LCSE Calculation

All LCSE values were computed as the difference in energy between the bound-state and global-minimum conformations (). An initial benchmark of AIMNet2-CPCM against QM used the previously reported bound-state and global-minimum conformations of the 6672 ligands in the Zhao-Set. The single-point energies at the B97-3c^48^ level using CPCM^49^ for these conformations were computed using ORCA 5.0.3.^50^ LCSE is sensitive to protonation and tautomeric state, so both were held identical between the bound-state and global-minimum end points of every ligand. The state assigned to each bound-state structure was carried unchanged into conformer generation and optimization of the unbound state, so LCSE reflects conformational reorganization at fixed ionization. Both end points are evaluated in CPCM­(water), so the reported LCSE is an internal strain referenced to aqueous solution. We adopt this as a defined convention, and it does not imply that the bound conformer occupies bulk water. The true environment of a bound ligand is a heterogeneous and often partially desolvated pocket. Computing the bound-state energy in CPCM­(water) provides a consistent aqueous reference for the strain, not a model of the pocket.

To compare against classical force fields, we computed strain with MMFF94 in two ways. Single-point strain uses MMFF94 energies of the AIMNet2-CPCM bound and global conformers without reoptimization. Reoptimized strain relaxes both end points on the MMFF94 surface: the bound conformer with all rotatable-bond dihedrals restrained to their deposited values, and the global conformer without restraints. Because the global end point is relaxed from the AIMNet2-CPCM minimum rather than from a full MMFF94 conformer search, the reoptimized value is a local force-field strain. To separate the effect of solvation from that of the force field, we also computed GAFF2 strain with AM1-BCC charges in vacuum and in the OBC2 generalized Born model, using OpenMM with openmmforce fields. These calculations were run as single points and with reoptimized end points, as for MMFF94, on a stratified subset of 50 ligands with 10 each at net charge 0, +1, −1, −2 and −3. All force-field values are provided in the SI data tables. To attribute strain to conformational change, we counted the rotatable-bond dihedrals that differ by more than 30 degrees between the bound and global conformers, using the deposited connectivity. Crystal-structure quality, namely resolution and the electron-density fit score EDIAm,^55^ was taken from the Tong and Zhao data set. As an independent higher-level reference for neutral ligands, we used LigBoundConf 2.0, whose strains were computed in vacuo at wB97M-D3­(BJ)/def2-TZVPPD. Per-ligand uncertainty was estimated as the standard deviation of the strain across the four AIMNet2-CPCM ensemble members. Each member was evaluated on both the bound and global conformers, so the part of the model error shared by the two end points cancels in their difference.

### Analysis of LCSE

Molecular properties were used to analyze LCSE profiles. The total number of rotatable bonds was obtained with Omega Toolkit^54^ since it is used for conformer generation to ensure consistency with the global-minimum search. The relationship between LCSE and the aromaticity of the ligands was studied using the proportion of the number of aromatic bonds () to the total number of bonds which were obtained with RDKit. Information on whether a ligand is annotated as a cofactor, and the class of the enzyme (if any) to which it is bound, was obtained with the programmatic access to PDBe data using the REST API.^56^ LCSE was obtained for all 7887 ligands, of which 304 are annotated as cofactors and 3340 occur in more than one PDB entry. Excluding the ligands which are identified as cofactors, we identified the class of the target enzyme for 5247 ligands. In the following text the set containing ligands with identified EC targets will be referred to as the *EC-Set*. 3D representations are rendered using Visual Molecular Dynamics^57^ (VMD).

## Results and Discussion

To test the accuracy of the AIMNet2-CPCM model, we performed multiple benchmarks. First, we computed solvation energies () for structures obtained from FreeSolv v0.51 database^58^ and compared them with the reference QM calculations. The gas-phase^44^ and CPCM AIMNet2 models (both at the B97-3c level) can be used together to compute solvation energies accurately (Figure 2a). Second, we computed single-point energies for the reference bound-state and global-minimum conformations of the Zhao-Set (see Supporting Information, Figure S1) and compared the predictions with the reference QM calculations. AIMNet2-CPCM reproduces the energies of different conformations of the same molecule, with an RMSE of 1.21 kcal/mol for bound-state and 1.81 kcal/mol for global-minimum conformers. In conjunction with this benchmark, we computed LCSE using AIMNet2-CPCM model for the reference conformations in the Zhao-Set and compared the results with the reference QM calculations (Figure 2b). The resulting LCSE values are reliable, with a coefficient of determination (*R* ^2^) of 0.9, an RMSE of 1.35 kcal/mol and an MAE of 0.94 kcal/mol. These benchmarks show that AIMNet2-CPCM provides reliable energies for different molecules and their conformations.

**Figure 2:**
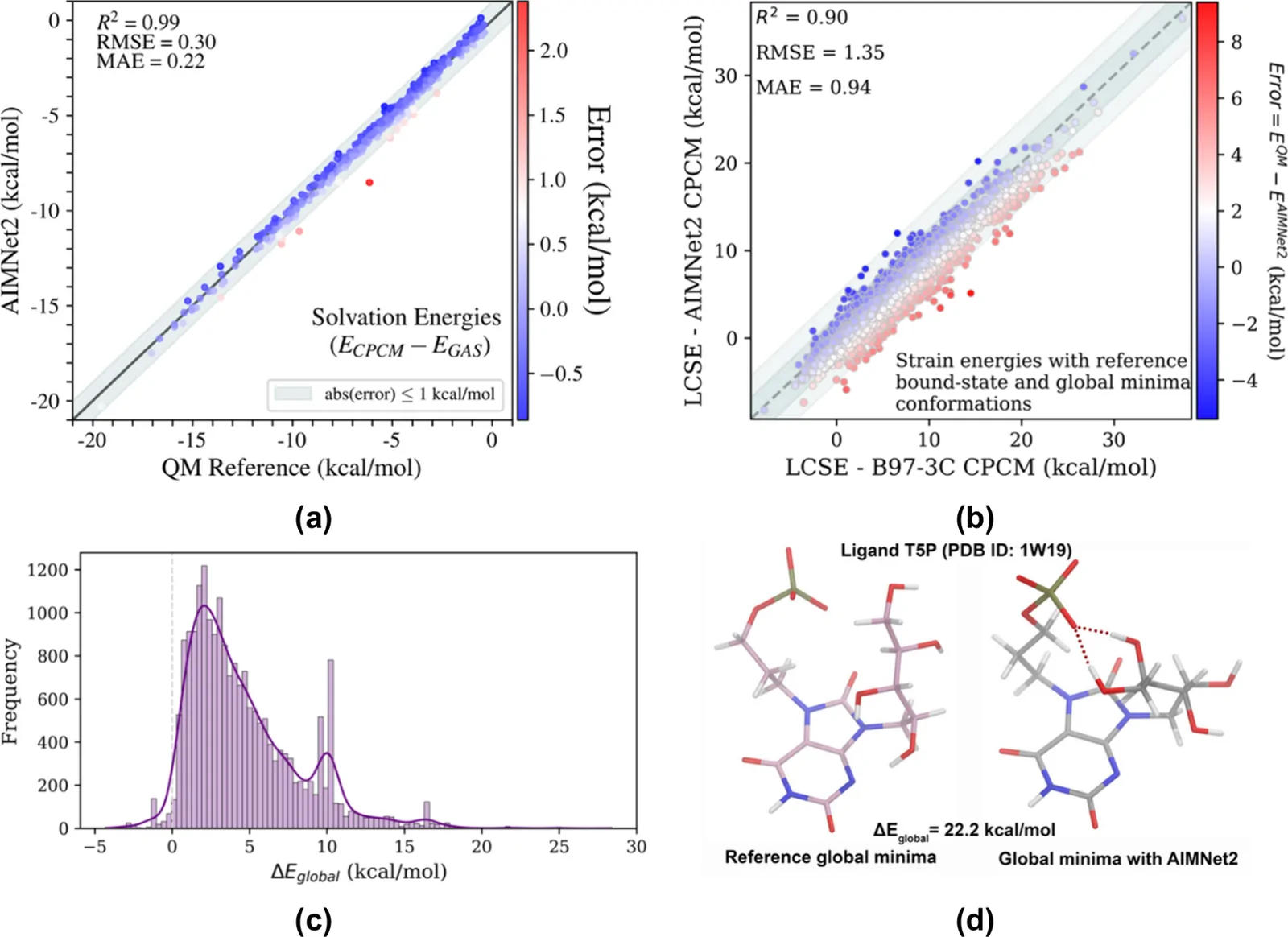
AIMNet2-CPCM model benchmarks. (a) Solvation energies () obtained with AIMNet2 gas^44^ and CPCM models at B97-3c level with respect to QM reference for FreeSolv v0.51. (b) LCSE computed with reference global and bound conformers using AIMNet2-CPCM model with respect to QM reference energies. (c) Energy difference between the reference global-minimum conformers and conformers obtained with AIMNet2-CPCM model. (d) The reference global-minimum conformation (left) of ligand T5P (PDB ID: 1W19) and the global-minimum conformation obtained with AIMNet2-CPCM model (right).

Up to this point all calculations on the LigBoundConf data set used the reference bound and global minima (Zhao-Set). These conformations were found with a classical force field (FF) from a small set of generated conformers, 500 for ligands with more than five rotatable bonds and 100 otherwise, which were then refined with QM calculations. To assess this procedure, which is popular for LCSE calculations, we compared, at the AIMNet2-CPCM level, the energies of the global minima located by AIMNet2-CPCM with the energies of the reference global-minimum conformers (Figure 2c).

For most ligands the AIMNet2-CPCM search located conformers lower in AIMNet2-CPCM energy than the reference minima. This is consistent with broader sampling, up to 5000 conformers per structure, combined with optimization on the same potential, whereas the reference was built with a classical FF and limited sampling. We emphasize that Figure 2c evaluates energies on the AIMNet2-CPCM surface. It therefore establishes that the extended search finds conformers the reference protocol missed, not that these conformers are lower in energy at the reference DFT level. Confirming the energetic ordering would require B97-3c/CPCM single-point or reoptimized energies for both sets of minima.

The AIMNet2-CPCM model on GPUs allowed these calculations at lower computational cost than QM while being less sensitive to the torsional errors of classical FFs. An example is depicted in Figure 2d. The presence of charged groups and multiple rotatable bonds in ligand T5P (PDB ID: 1W19) illustrates the effect of a broader conformer search. AIMNet2-CPCM located a minimum where the tetrahydroxypentyl group donates H-bonds to the oxygen atoms on the propyl dihydrogen phosphate group, while the reference minimum contains no such interaction.

For a polyanionic ligand such as T5P, the CPCM continuum screens intramolecular electrostatics imperfectly and can overstabilize collapsed conformers that donate intramolecular hydrogen bonds. In bulk water the same groups would be solvated by explicit water molecules and counterions. If so, the located minimum is stabilized artificially and the LCSE of this ligand is overestimated. This example should therefore be read as an illustration of charge-aware sampling rather than as a validated strain magnitude. A ML potential that cannot handle charged species, or one trained on gas-phase reference data, would be prone to locating a different minimum and a correspondingly different LCSE. Taken together, these results indicate that AIMNet2-CPCM offers a practical, solvation-aware alternative to popular conformer-search approaches and gas-phase ML potentials for LCSE, subject to the DFT-level confirmation noted above.

Having validated AIMNet2-CPCM against reference strains, we applied it across the full library. We computed LCSE for 7887 PDB ligands as the AIMNet2-CPCM energy difference between the torsion-constrained bound conformer and the global minimum located in continuum water. The resulting distribution is right-skewed, with a median of 4.0 and a mean of 5.4 kcal/mol (Figure S2). Strain increases with net charge, and it does so asymmetrically. The median is 2.7 kcal/mol for neutral ligands, 6.3 kcal/mol at charge +2 and 9.1 kcal/mol at charge −2. At charge −3 and −4 the medians are 12.2 and 17.7 kcal/mol. These ligands form the polyanionic phosphate and nucleotide tail, where continuum solvation is least reliable. We therefore report these high-charge magnitudes as upper bounds (see Limitations). The quantitative charge trend should be read as well determined through net charge −2.

A classical force field reproduces the solvated strain of neutral ligands but not of charged ones. MMFF94 single-point energies on the same conformers agree with AIMNet2-CPCM to a median absolute difference of 2.2 kcal/mol for neutral ligands, with no systematic bias. For charged ligands the gas-phase force field assigns larger strain, by a median of 11.6 kcal/mol at net charge −2 and 20.3 kcal/mol at −3 (Figure S15). Reoptimizing both end points on the MMFF94 surface does not close the gap; it widens it. The reoptimized force-field strain exceeds the AIMNet2-CPCM value by a median of 1.3 kcal/mol for neutral ligands, 19.6 kcal/mol at charge −2 and 29.2 kcal/mol at charge −3. The reason is that the unrestrained global end point relaxes further into the compact, intramolecularly hydrogen-bonded or salt-bridged conformers that unscreened electrostatics favor. Rank correlation with AIMNet2-CPCM stays moderate throughout, with Spearman coefficients of 0.56 to 0.78 across charge classes, so the force field orders ligands reasonably but misplaces the charged ones on the absolute scale. The divergence is a solvation effect rather than a force-field artifact. On a stratified subset of 50 ligands, GAFF2 in vacuum behaves like MMFF94 and overestimates the reoptimized strain by a median of 23.1 kcal/mol at charge −2 and 21.6 kcal/mol at −3. Adding the OBC2 generalized Born model removes this bias almost entirely: the median bias drops to −1.9 and −1.7 kcal/mol, and the median absolute differences from AIMNet2-CPCM are 3.4 and 3.8 kcal/mol (Figure S16). Solvent screening of the unbound reference is therefore the dominant correction for charged ligands. Any method that omits it, whether a force field or a gas-phase ML potential, inflates their strain. The residual differences of 1 to 4 kcal/mol between solvated GAFF2 and AIMNet2-CPCM are of the size expected from torsional parametrization and from the local nature of the force-field reoptimization. They are where a QM-quality potential adds accuracy beyond a solvated force field.

The neutral strains agree with an independent, higher-level reference. LigBoundConf 2.0 reports strains computed in vacuo at wB97M-D3­(BJ)/def2-TZVPPD. For the 2694 neutral ligands shared with that set, AIMNet2-CPCM ranks strain consistently, with a Spearman coefficient of 0.63, and predicts solvated strains on average 1.6 kcal/mol below the gas-phase reference (Figure S3), as expected when solvation is included.

The strain estimates are not an artifact of crystal quality. Using the resolution and electron-density fit (EDIAm) reported for every ligand, we find that LCSE shows no meaningful correlation with either. The Spearman coefficients are 0.05 for resolution and 0.06 for EDIAm (Figure S4). The median strain rises only slightly, from 3.7 kcal/mol below 1.5 Å to 4.2 kcal/mol between 2.0 and 2.5 Å. Poorly resolved structures do not carry systematically higher strain, which argues against the concern that refinement error in the constrained bound geometry dominates the values.

Strain accumulates across many torsional changes rather than a single large one. LCSE correlates with the number of rotatable-bond dihedrals that change by more than 30 degrees between the bound and global conformers, with a Spearman coefficient of 0.57 (Figure S5). It correlates only weakly with the largest single change (0.11). The median strain rises from 0.2 kcal/mol when no torsion changes to 5.6 kcal/mol when five or more change. Strain is therefore distributed over many small torsional adjustments rather than one large rotation, which directs design toward reducing overall flexibility rather than fixing a single rotor.

The four-member AIMNet2-CPCM ensemble provides a per-ligand uncertainty on each LCSE, taken as the standard deviation of the strain across the members. This spread is small. The median is 0.48 kcal/mol, and 88% of ligands fall below 1 kcal/mol, so the reported strains do not depend on the choice of ensemble member. The uncertainty grows mainly with conformational flexibility: it increases with the number of torsions that change between the bound and global conformers (Spearman 0.51). The dependence on net charge is weak (Spearman 0.24); the median rises from about 0.4 kcal/mol for neutral and singly charged ligands to about 0.9 kcal/mol at charge −4 (Figure S6). This spread reflects the internal consistency of the ensemble rather than the physical accuracy of the strain, a distinction we return to in the Limitations.

### LCSE Distribution with Respect to Molecular Properties

We studied the distribution of LCSEs of 7887 ligands in LigBoundConf^13^ with respect to molecular properties of each ligand (Figure 3). These distributions elaborate the charge trend reported above and extend it to molecular size, flexibility and aromaticity. Distribution of LCSEs with respect to the net charge of molecules is shown in Figure 3a. Neutral and singly charged ligands mostly have LCSE below 10 kcal/mol, with a 75th percentile (P75) of 6.2 kcal/mol (). LCSE increases with the magnitude of the net charge, as seen for ligands with net charges of ±2 and ±3 (Figure S7). Similar results were observed when molecules that are not cofactors or molecules which bind to single target are considered separately (Figures S8 and S9). Among cofactors, the distribution for species with a net charge of −2 is bimodal, with modes near 4.3 and 12.3 kcal/mol (Figure S10). The same two modes recur in the size, flexibility and aromaticity distributions of cofactors below. We show in the multitarget section that they arise because a few cofactors that occur in hundreds of entries adopt two families of bound geometries. Highly charged ligands can afford larger strain because the pocket repays it with strong intermolecular interactions, such as salt bridges and metal coordination of phosphate groups, that neutral ligands cannot form. In addition, the solvated global minimum of a polyanion is extended to maximize solvent exposure of its charges, whereas the bound pose is often compact, so the conformational change itself is larger. Similar conclusions were reported for ligands with and without formally charged atoms.^13^ The property-level trends reported here, higher strain with higher net charge and with more rotatable bonds, confirm relationships already described by Tong and Zhao and by related studies. What is new is their application to two analyses that earlier work had not pursued at this scale: the distribution of LCSE across enzyme classes and across multitarget binders, presented in the following sections.

**Figure 3:**
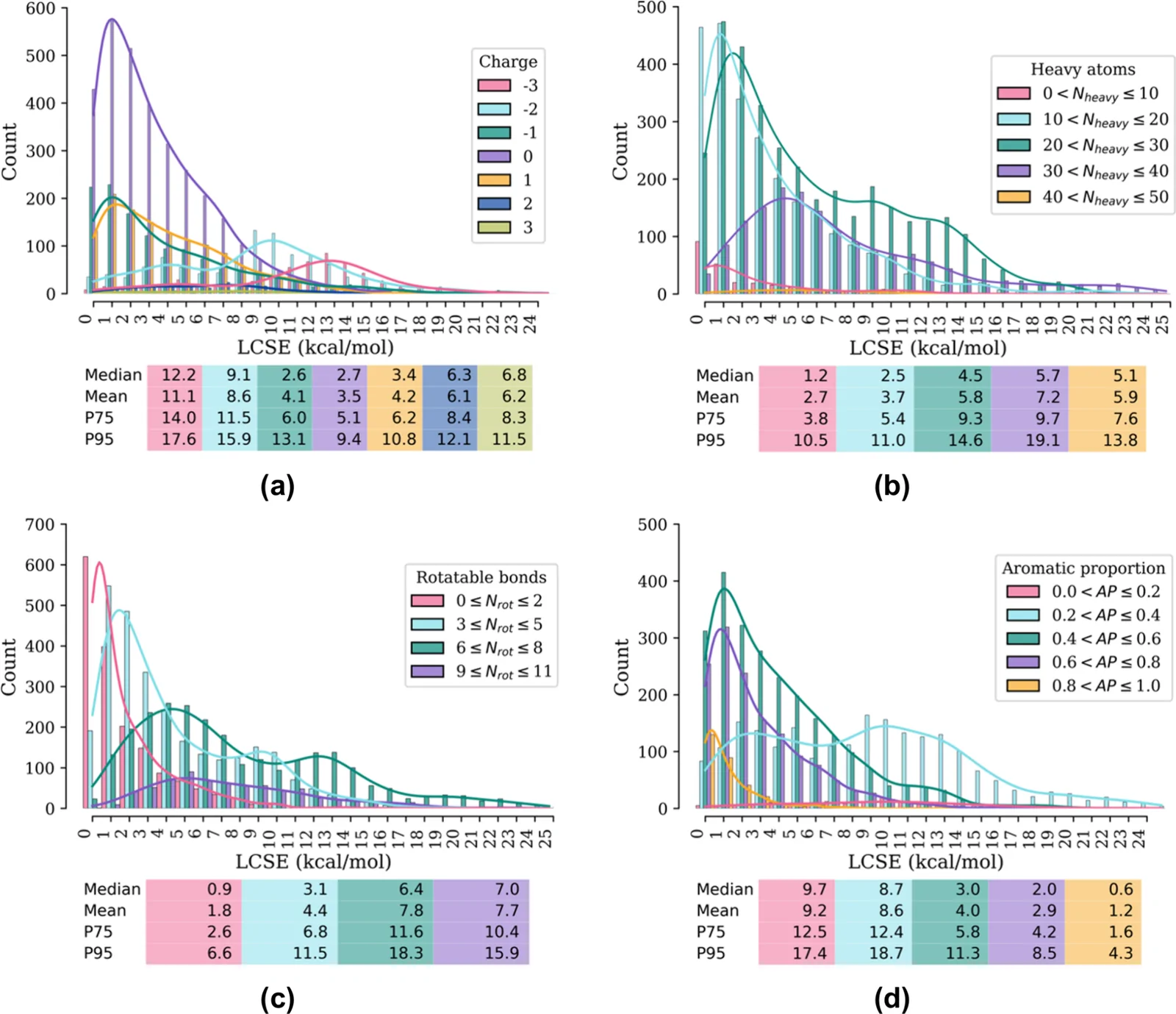
Conformational ligand strain energy profiles computed with AIMNet2-CPCM model. Strain energy distribution with respect to (a) total charge, (b) number of heavy atoms, and (c) number of rotatable bonds and (d) aromatic proportion, as obtained with the Omega Toolkit.

The distributions of LCSE against the number of heavy atoms, the number of rotatable bonds, and aromatic proportion were also analyzed. Heavy-atom count shifts the most populated LCSE values (Figure 3b). Ligands with fewer than 20 heavy atoms mostly have LCSE below 6 kcal/mol, with medians below 3 kcal/mol. Ligands with 20 to 30 and 30 to 40 heavy atoms mostly stay below 10 kcal/mol, with medians of 4.5 and 5.7 kcal/mol, respectively. The largest ligands, with more than 40 heavy atoms, show lower LCSE again, but only 62 of them are present, too few to support a general conclusion. Among cofactors, compounds with 30 to 40 heavy atoms show a bimodal distribution, mirroring the one seen for net charge (Figure S10). Ligand size therefore influences LCSE, but other molecular properties also contribute, as shown by the wide LCSE range of ligands with 20 to 30 heavy atoms.

Consistent with the torsional-accumulation result above, LCSE increases with the number of rotatable bonds (Figure 3c). Ligands with fewer than 2 rotatable bonds mostly have LCSE below 3 kcal/mol. Ligands with 3 to 5 rotatable bonds show a bimodal distribution, with 75% of the population below 6.8 kcal/mol. A bimodal distribution also appears for ligands with 6 to 8 rotatable bonds, most of which fall below 11.6 kcal/mol. Ligands with 9 to 11 rotatable bonds are few, but their mean, median and P75 are similar to those of the 6 to 8 group. Aromatic proportion (AP) shows the opposite trend: ligands with higher AP have lower LCSE (Figure 3d). Ligands with AP above 0.4 predominantly have LCSE below 6 kcal/mol, with a P75 below 5.8 kcal/mol, while ligands with AP between 0.2 and 0.4 show a bimodal distribution. Among cofactors, LCSE is again bimodal for both rotatable-bond count and aromatic proportion, with the same modes near 4.3 and 12.3 kcal/mol. Because high-AP molecules tend to have fewer rotatable bonds, their low LCSE parallels that of molecules with few rotatable bonds. More degrees of freedom, whether from additional rotatable bonds or lower aromatic proportion, raise LCSE: as flexibility grows, more torsions can deviate from their solution-phase minima, and the strain accumulates over these deviations (Figure S5).

We also analyzed the compounds with different numbers of phosphate groups to isolate the effect of the phosphate chain length. The distribution of these compounds can be found in Figure S11. The P75 of LCSE for AMP, ADP and ATP is 10.5, 14.4, and 23.6 kcal/mol, respectively. Similar patterns are observed for uridine-5′-mono/di/triphosphate (**U5P**/**UDP**/**UTP**), cytidine-5′-mono/di/triphosphate (**C5P**/**CDP**/**CTP**), and guanosine mono/di/triphosphate (**5GP**/**GDP**/**GTP**). While the number of entries of diphosphate and/or triphosphate derivatives of these compounds are not as large as those of the monophosphate derivatives in our set, the similarities in the LCSE distributions indicate that LCSE increases with the number of phosphate groups. This follows from the additional degrees of freedom and the additional charge of each phosphate. The high-charge tail of the distribution should be read with an important caveat. Sixteen of the 17 ligands with LCSE above 25 kcal/mol are phosphate and nucleotide species with net charge −2 to −4 (Table S1), and their absolute values are best treated as upper estimates for the reasons given in the Limitations. The exception is MHA in PDB entry 1IX1, a 13-heavy-atom ligand with net charge −1, whose value most likely reflects its deposited torsions rather than a physical penalty.

### LCSE Distribution with Respect to Enzyme Class (EC)

Previous large-scale studies analyzed strain against molecular properties only.^13–15,18,19,21,47^ Because AIMNet2-CPCM makes thousands of solvated strain calculations routine, we can also ask how strain distributes across target classes. To do so, we excluded the compounds in LigBoundConf^13^ that are annotated as cofactors and selected ligands whose targets have an enzyme class assigned in PDBe.^56^ The remaining entries, the EC-Set, were split into subsets according to their target EC.

The distributions of LCSE for the ligands in the EC-Set can be found in Figure 4 and the distribution of LCSE with respect to enzyme subclasses can be found in Figure S13. Most ligands in the EC-Set target transferases (∼2500 ligands) and hydrolases (∼1300 ligands) (Table S2). Ligands of transferases, oxidoreductases and hydrolases have median LCSE below 4 kcal/mol. The median increases for ligands of isomerases (6.3 kcal/mol), ligases (7.9 kcal/mol) and lyases (7.5 kcal/mol), and the distributions for isomerases and lyases are bimodal. The majority of these ligands have LCSE lower than ∼11 kcal/mol (for isomerases, and for lyases). For ligases the LCSE is distributed more evenly than for the most populated classes, with mean and median near 8 kcal/mol and P75 near 11 kcal/mol. These class-level differences should be read with caution. The classes with elevated strain, in particular ligases, lyases and isomerases, disproportionately bind ATP and other charged nucleotide phosphates, the same high-charge chemotypes that carry high strain in the property-level analysis above. The observed trends therefore reflect the chemotypes that each enzyme class tends to bind rather than a causal property of the enzyme class itself.

**Figure 4:**
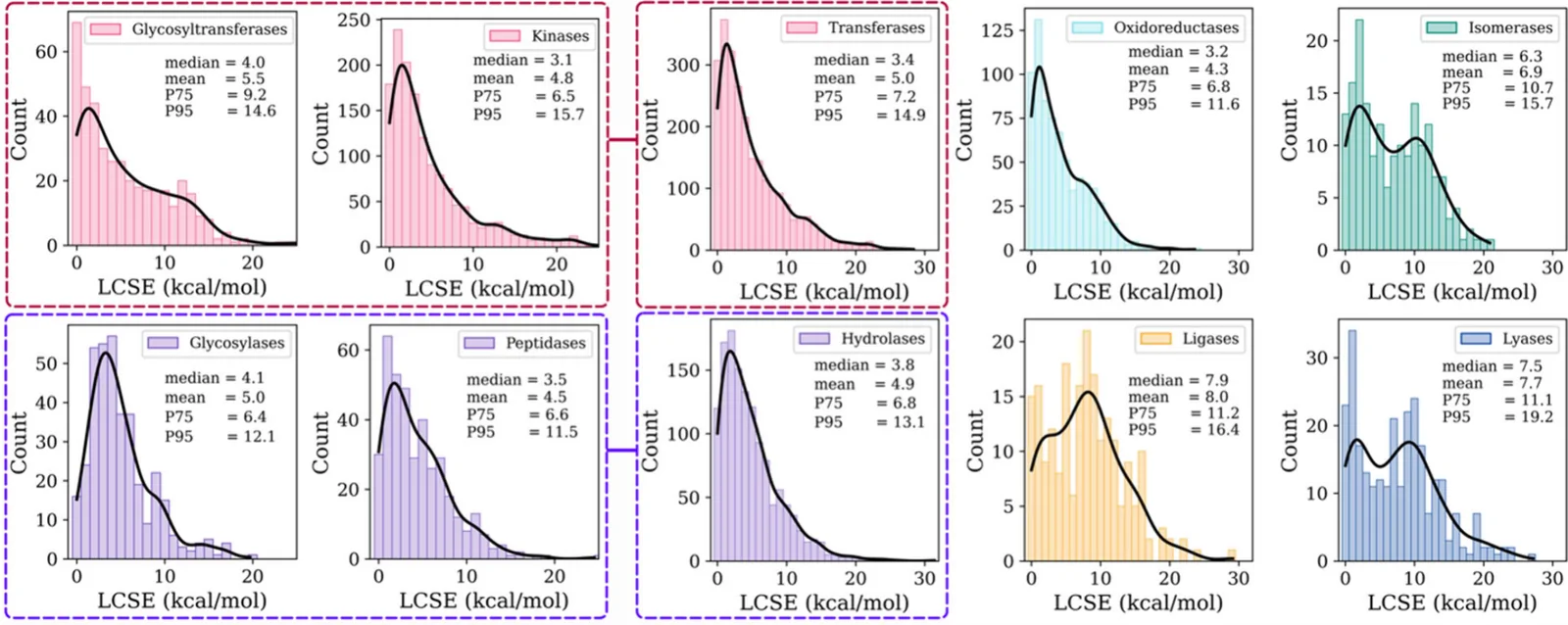
LCSE profile for ligands that are not identified as cofactors with respect to enzyme class. Only enzyme classes with more than 20 entries are plotted.

The predominant transferase subclass in the EC-Set is the kinases, which transfer phosphorus-containing groups from a phosphate donor. Kinase ligands mostly have LCSE below 6.5 kcal/mol, consistent with the conserved ATP-site fold and the flat, aromatic hinge binders that dominate this class.^59–62^ The second most populated subclass is the glycosyltransferases (GTases), which attach sugars to sugars, lipids, proteins and nucleic acids; despite their functional diversity, most belong to one of 2-fold superfamilies (GT-A and GT-B).^63,64^ GTase ligands have a mean LCSE of 5.5 kcal/mol and a median of 4 kcal/mol. Their P75 of 9.2 kcal/mol is higher than that of kinase ligands and reflects the chemical diversity of their substrates.

Most hydrolase ligands in the EC-Set target peptidases (391 ligands) or glycosylases (371 ligands). Peptidases, which cleave peptide bonds and are classified into families and clans by the MEROPS system,^65,66^ are the targets of many drug discovery programs, most recently the coronavirus main protease.^67^ Most peptidase ligands have LCSE below 7 kcal/mol () and a 95th percentile of 11.5 kcal/mol. Most glycosylase ligands have LCSE below 6.5 kcal/mol () with a median of 4.1 kcal/mol and a mean of 5.0 kcal/mol; DNA glycosylases, which recognize and excise damaged bases, are targets of several drug discovery programs.^68,69^

### LCSE Distribution of Binders with Multiple Targets

Ligands that bind more than one target are the second analysis that per-ligand studies have not addressed. Conformational strain has been linked to selectivity. Second-generation anaplastic lymphoma kinase (ALK) inhibitors such as brigatinib adopt a U-shaped conformation in the ALK kinase domain. A bridging group that holds this shape contributes to selectivity against other kinases, which would have to pay strain to bind the same conformation.^70–72^ Rigidification also removes the entropic cost of immobilizing rotors, so a preorganized ligand gains on both terms, and the two cannot be separated by strain alone. Conversely, polypharmacological drugs designed to bind several targets^73–76^ must tolerate an acceptable strain at each of them. Both design goals require knowing how the strain of one ligand varies across the targets that bind it, which motivated us to analyze the LCSE distributions of multitarget compounds in the LigBoundConf data set.

Ligands that appear in multiple PDB entries can span a wide range of LCSE. Among the ligands present in more than 20 entries (Figure S12), some vary little across their targets, such as inosinic acid, whereas others differ by more than 10 kcal/mol, such as adenosine-5′-diphosphate, or separate into two distinct modes. Flavin mononucleotide (FMN), which acts as a coenzyme and metabolite, is the clearest such case (Figure 5). Across its 186 entries the LCSE distribution is bimodal, with modes near 4.4 and 12.2 kcal/mol. These entries bind oxidoreductases, transferases, translocases and isomerases. Aligning the deposited bound conformations shows that the low-strain conformations, those with, share a common isoalloxazine ring orientation, whereas the number of accessible orientations grows with LCSE and the ribitol-phosphate moiety stays conformationally diverse throughout. The two strain modes therefore correspond to distinct bound geometries selected by different pockets, so the strain of FMN reflects the geometry enforced by each binding site rather than a fixed molecular property. Because FMN, FAD, NAD and the nucleoside phosphates together account for a large share of the cofactor entries, this target dependence is also the origin of the bimodal cofactor distributions in Figure 3 and Figure S10.

**Figure 5:**
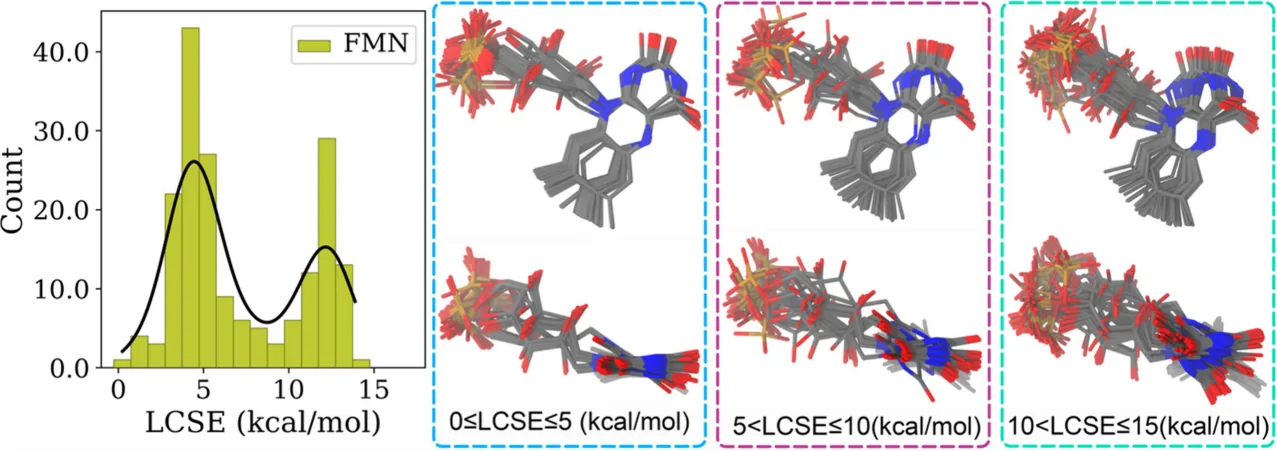
LCSE distribution of flavin mononucleotide (FMN) and three-dimensional representations of aligned bound-state conformations grouped with respect to LCSE.

The target dependence that FMN exemplifies can be quantified across the data set. Among the 48 ligands present in at least ten PDB entries, the within-ligand range of LCSE across targets has a median of 7.5 kcal/mol, and one-third of these ligands span more than 10 kcal/mol (Figure 6). The spread grows with conformational flexibility, with a Spearman coefficient of 0.44. It also grows with the range in resolution across the entries of a ligand (Spearman 0.40), so part of the apparent variation reflects differences in structure quality rather than in strain. Resolution does not bias strain across the library (Figure S4), but for a single ligand the spread across entries partly reflects how well each pose is defined. The within-ligand range is therefore an upper bound on the true target dependence. The dependence on net charge is weak (Spearman 0.33). A single strain value is therefore not a transferable descriptor of a ligand. It is a property of the specific bound pose, and its reliability is bounded by the quality of the structure that defines that pose.

**Figure 6:**
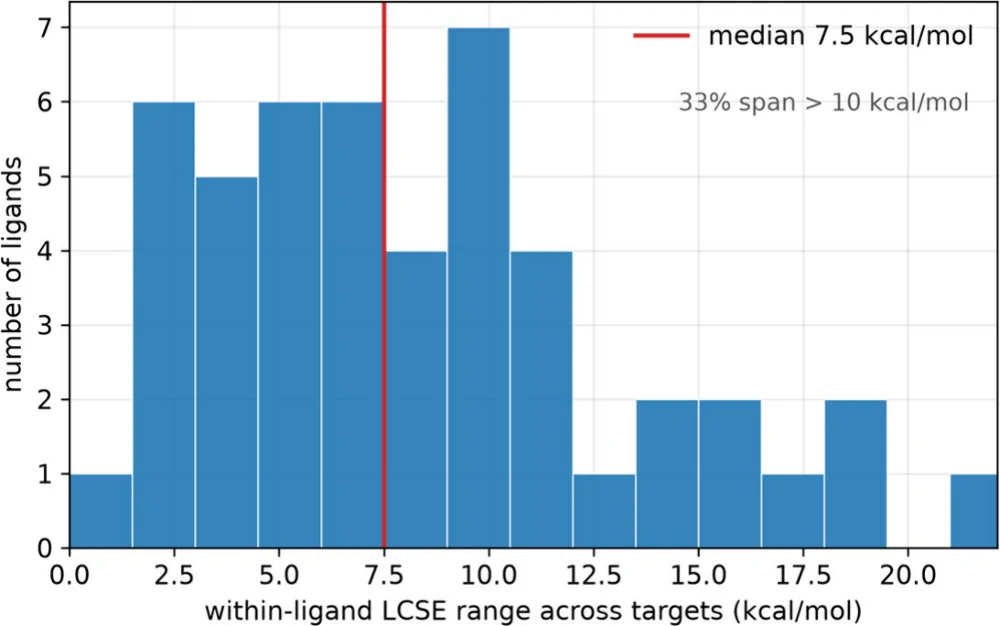
Ligand strain varies across targets. Distribution of the within-ligand LCSE range across its targets for the 48 ligands present in at least ten PDB entries; the median range is 7.5 kcal/mol.

### Limitations

Several factors bound the interpretation of the LCSE values reported here. First, both the reference and the AIMNet2-CPCM model rely on continuum solvation, which becomes less reliable as the net charge of the ligand grows.^77^ For the polyanionic phosphate and nucleotide species^78^ that dominate the high-strain tail, CPCM can overstabilize compact intramolecularly hydrogen-bonded conformers relative to an explicit-solvent treatment, so their strain is likely overestimated. Second, the bound-state energy is derived from a crystallographic pose with torsions held at their deposited values. Refinement error in the coordinates propagates into the strain, and low-resolution or poorly fit structures can inflate individual LCSE values. Third, the reference level of theory, B97-3c with CPCM, is a low-cost composite method. Its conformational energy differences for flexible charged molecules can carry errors of 1 to 2 kcal/mol, comparable to the median strain. For these reasons the LCSE values are best treated as distributions and relative rankings across chemotypes and target classes rather than as exact per-ligand penalties. Individual absolute values, in particular in the high-charge regime, should be read as estimates.

Finally, the per-ligand ensemble spread reported above measures the internal consistency of the four networks, not the accuracy of the underlying B97-3c/CPCM description. The members share the same training target, so the systematic continuum-solvation error on polyanions is common to all of them and largely cancels in their spread. A small ensemble uncertainty on the high-charge tail is therefore not evidence that those individual strain values are physically accurate. We also evaluated the bound and global-minimum conformers of the polyanionic ligands with an implicit real-solvent model (openCOSMO-RS)^79^ as an alternative to CPCM. It is not parametrized for multiply charged anions and returns unphysical negative strain for them. It therefore cannot resolve the high-charge tail, and we retain the CPCM values as upper bounds.

## Conclusion

Ligand conformational strain is routinely estimated from a single gas-phase energy minimum, and we show that this shortcut misestimates the strain of charged drug-like ligands. AIMNet2-CPCM removes the artifact at screening scale. Trained to B97-3c energies and forces in a continuum solvent, it treats neutral and charged species on the same footing and reproduces reference quantum-mechanical strains for drug-like ligands outside its training set. Because inference is GPU-batched, a solvated global-minimum search that evaluates millions of conformers becomes practical across an entire screening library. Applied to 7887 PDB ligands, the solvated strain rises with net charge. The trend is well determined through net charge −2 and is reported as an upper bound for the −3 and −4 polyanion tail, where continuum solvation is least reliable. Gas-phase force fields overstate the strain of charged ligands, and a continuum-solvated force field recovers most of the correction. Solvation of the unbound reference, not force-field quality, is therefore the dominant error. The resulting strains behave as robust aggregate quantities. They are insensitive to crystal resolution and to the choice of ensemble member, and they accumulate across many small torsional adjustments rather than a single large rotation.

Operating at screening scale also let us ask a question that per-ligand quantum studies could not: does a ligand carry a single characteristic strain? It does not. Profiling strain across enzyme classes and across ligands that bind more than one target, two analyses that earlier works had not addressed at this scale, shows that the same chemical species can strain very differently depending on the pocket that binds it. Among the 48 ligands present in at least ten PDB structures, the within-ligand strain spans a median of 7.5 kcal/mol across targets, and flavin mononucleotide resolves into two distinct strain modes across its bound poses. Differences between enzyme classes should be read with care. The high-strain classes disproportionately bind charged nucleotide phosphates, so the pattern reflects the chemotypes each class tends to bind rather than a property of the class itself. The broader lesson is that ligand strain is a property of a specific bound pose and target rather than a transferable molecular descriptor, and a single strain value should not be assigned to a compound before its bound conformation is known.

The workflow supports several applications that we frame as prospects rather than as results established here. Realizing them will require validation outside the present scope, in particular per-ligand uncertainty tied to input structure quality and protonation state, and comparison against explicit-solvent references for highly charged ligands. Further development could extend the training data to additional solvent environments. It could also add the conformational entropy term, which LCSE omits and which rigidification lowers alongside strain, through conformer populations or vibrational analysis. With those pieces in place, solvation- and charge-aware strain estimates could flag high-strain poses in structure-based virtual screening,^80^ rank candidate scaffolds by how closely their unbound minimum approaches a target bioactive conformation, track how strain evolves as a chemical series is elaborated, and enter docking and free-energy pipelines as a routine, solvation-aware filter on ligand conformational strain.

## Supplementary Material



## Data Availability

The data and code supporting this study are openly available at https://github.com/isayevlab/ligand-strain-aimnet2 (documentation at https://isayevlab.github.io/ligand-strain-aimnet2/). The repository contains the bound-state and global-minimum conformers of all 7887 ligands with their AIMNet2-CPCM energies and a per-ligand master table with LCSE, net charge, descriptors, PDBe annotations and force-field strain. It also contains the MMFF94 and GAFF2/OBC2 comparison tables, the timing benchmarks, an ASE interface with a reference implementation of the strain protocol, and the analysis notebook and scripts that generate every figure and table of this work. The AIMNet2-CPCM model weights will be released through the same repository upon publication and are available from the corresponding author before then. Reference structures are from the LigBoundConf data set of Tong and Zhao; the independent neutral reference is LigBoundConf 2.0.

## Notes

Single-point energy benchmarks on the Zhao-Set (Figure S1); distribution of solvated LCSE (Figure S2); comparison with the LigBoundConf 2.0 reference (Figure S3); dependence on crystal quality (Figure S4) and on torsional change (Figure S5); ensemble uncertainty (Figure S6); LCSE distributions by net charge, for noncofactors, single-target binders and cofactors (Figures S7 to S10); phosphate-containing ligands (Figure S11); multitarget ligands (Figure S12); enzyme subclasses (Figure S13); conformer generation statistics (Figure S14); MMFF94 strain with single-point and reoptimized end points (Figure S15, Table S4); GAFF2 strain in vacuum and in OBC2 implicit solvent (Figure S16, Table S5); ligands with LCSE above 25 kcal/mol (Table S1); enzyme classes in the EC-Set (Table S2); computational cost (Table S3); forward-pass throughput versus batch size (Table S6) (PDF)
