# Supplementary material for: Efficient and Accurate Ligand Strain Calculations in Solution with the AIMNet2 Neural Network Potential
Source: J Chem Inf Model. 2026 Oct 21;66(21):14012–24. doi: 10.1021/acs.jcim.6c02468 (PMC13753510; doi:10.1021/acs.jcim.6c02468)
Supplement: Supplementary file 1 [file ci6c02468_si_001.pdf]

SUPPORTING INFORMATION
FOR

EFFICIENT AND ACCURATE LIGAND STRAIN CALCULATIONS IN SOLUTION WITH THE AIMNET2 NEURAL NETWORK POTENTIAL

Hatice Gokcan[a], Olexandr Isayev[*a,b]

*Corresponding author. E-mail: olexandr@olexandrisayev.com
a Department of Chemistry, Mellon College of Science, Carnegie Mellon University, Pittsburgh, PA 15213, USA
b Department of Materials Science and Engineering, Carnegie Mellon University, Pittsburgh, PA 15213, USA

1. Conformational energies with AIMNet2

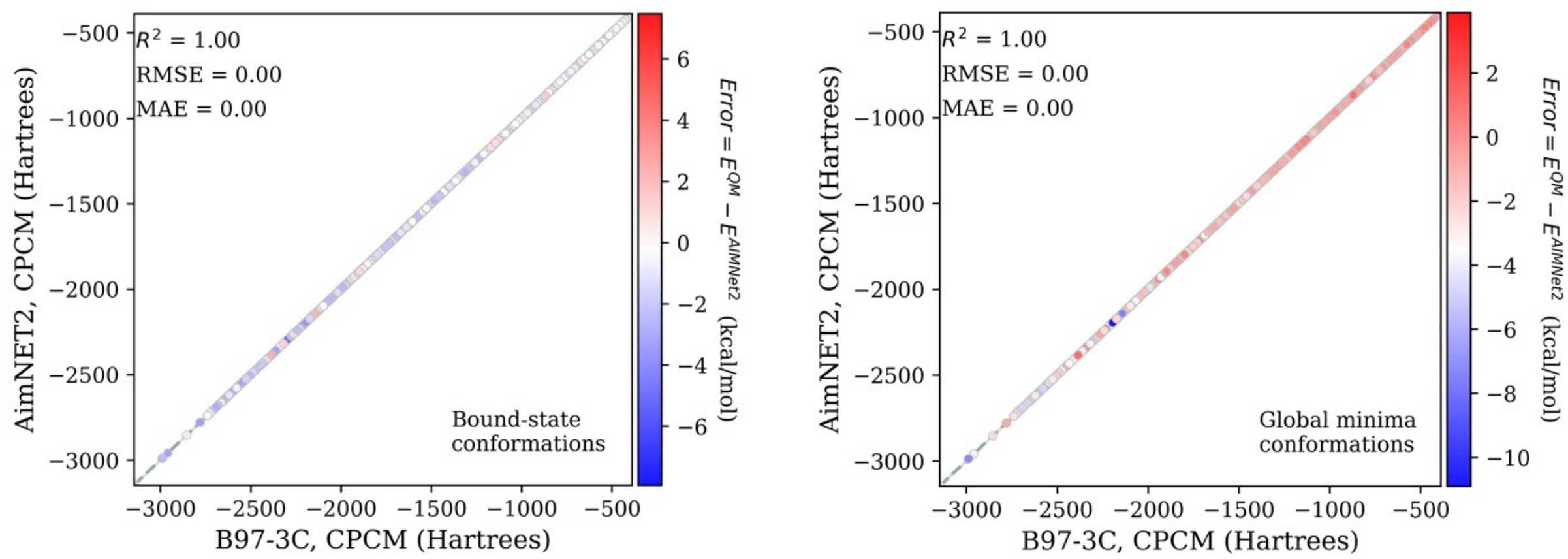

(a) **(b)**

Figure S1. Instantaneous energies of bound-state and global conformations from reference data containing ~6672 ligands with LCSE computed with QM calculations.

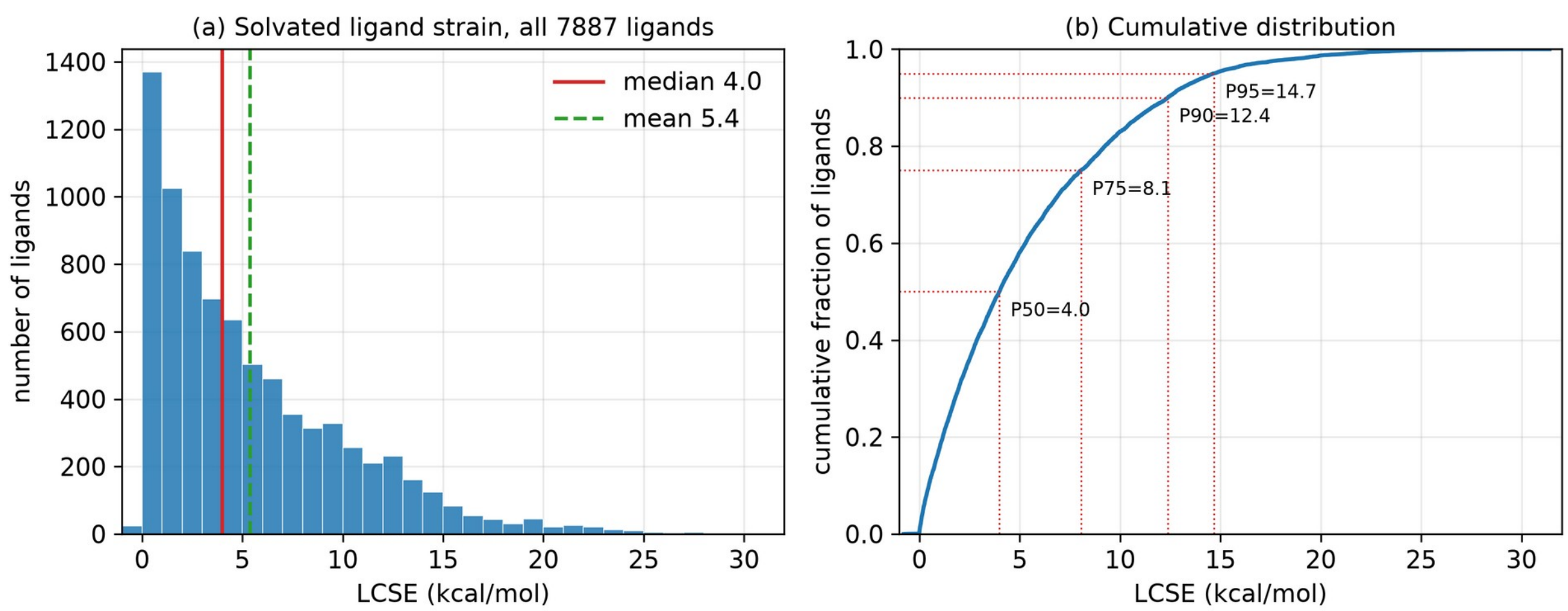

Figure S2. Distribution of solvated ligand conformational strain energy (LCSE) across all 7887 PDB ligands. (a) Histogram of LCSE, with the median (4.0 kcal/mol, solid line) and mean (5.4 kcal/mol, dashed line) marked; the distribution is right-skewed. (b) Cumulative distribution of LCSE; the 50th, 75th, 90th, and 95th percentiles fall at 4.0, 8.1, 12.4, and 14.7 kcal/mol, respectively. LCSE is the AIMNet2-CPCM energy difference between the torsion-constrained bound conformer and the solvated global minimum, averaged over the four-member ensemble.

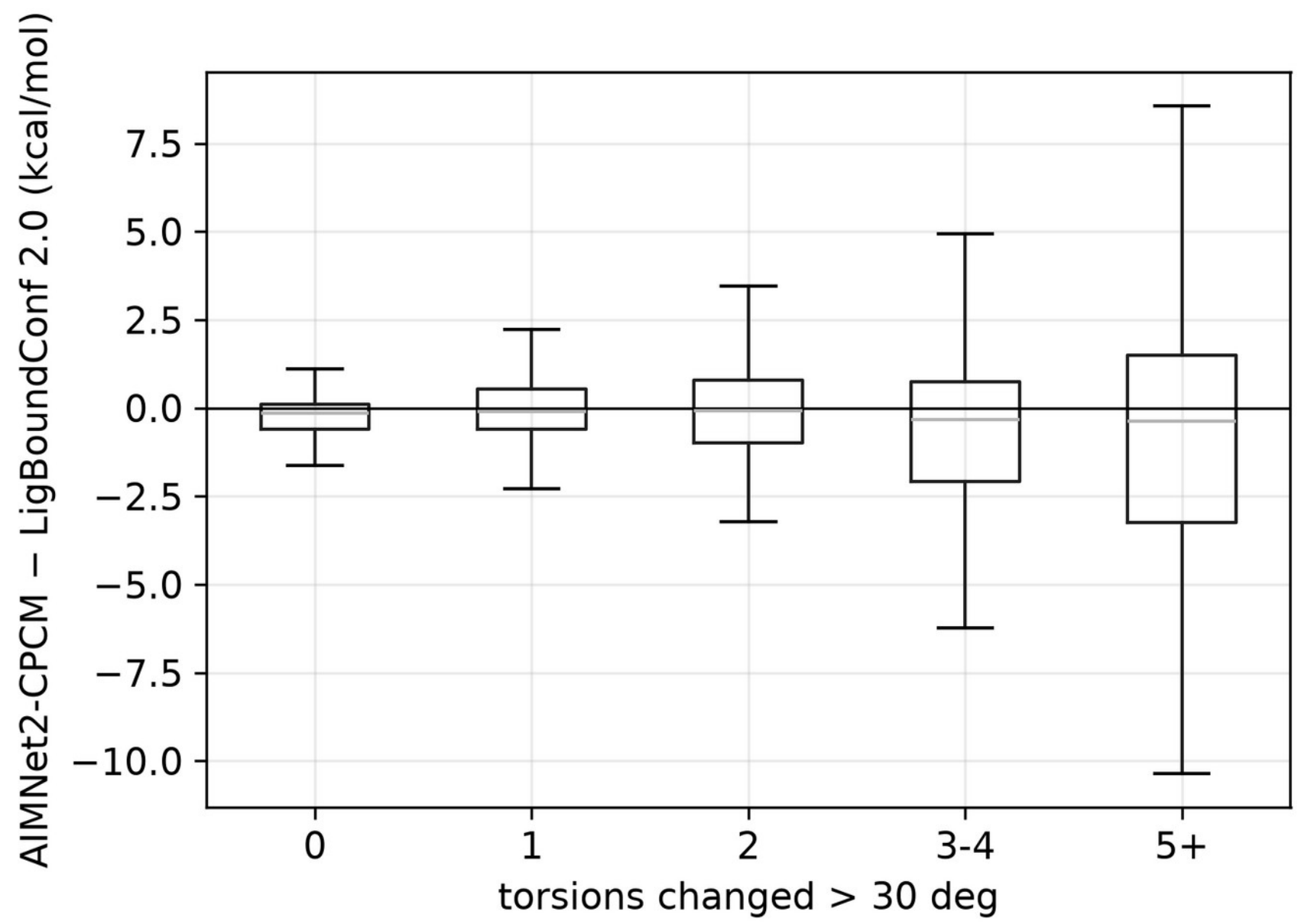

Figure S3. AIMNet2-CPCM solvated strain minus the LigBoundConf 2.0 in-vacuo higher-level reference (wB97M-D3(BJ)/def2-TZVPPD; neutral ligands only), binned by the number of rotatable-bond dihedrals that change by more than 30 degrees between the bound and global conformers. The solvated strains sit on average about 1.6 kcal/mol below the gas-phase reference, as expected when solvation is included.

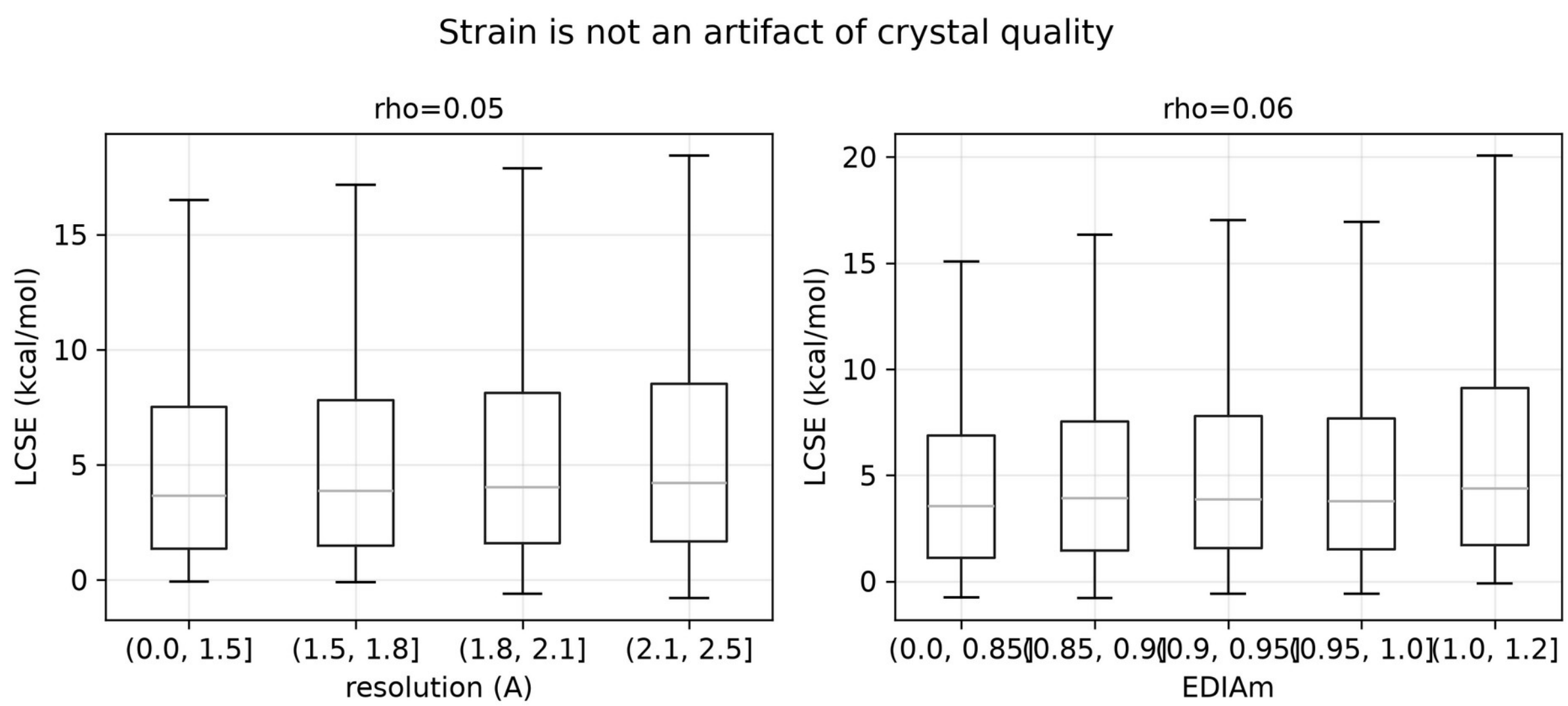

Figure S4. Ligand strain is not an artifact of crystallographic quality. Conformational ligand strain energy (LCSE) for all 7887 ligands, binned by (a) crystallographic resolution and (b) the electron-density fit of the bound ligand (EDIAm). Neither variable shows a meaningful monotonic trend (Spearman 0.05 and 0.06, respectively); the median LCSE rises only slightly, from 3.7 kcal/mol below 1.5 A to 4.2 kcal/mol between 2.0 and 2.5 A. Boxes span the interquartile range, whiskers the 5th-95th percentiles.

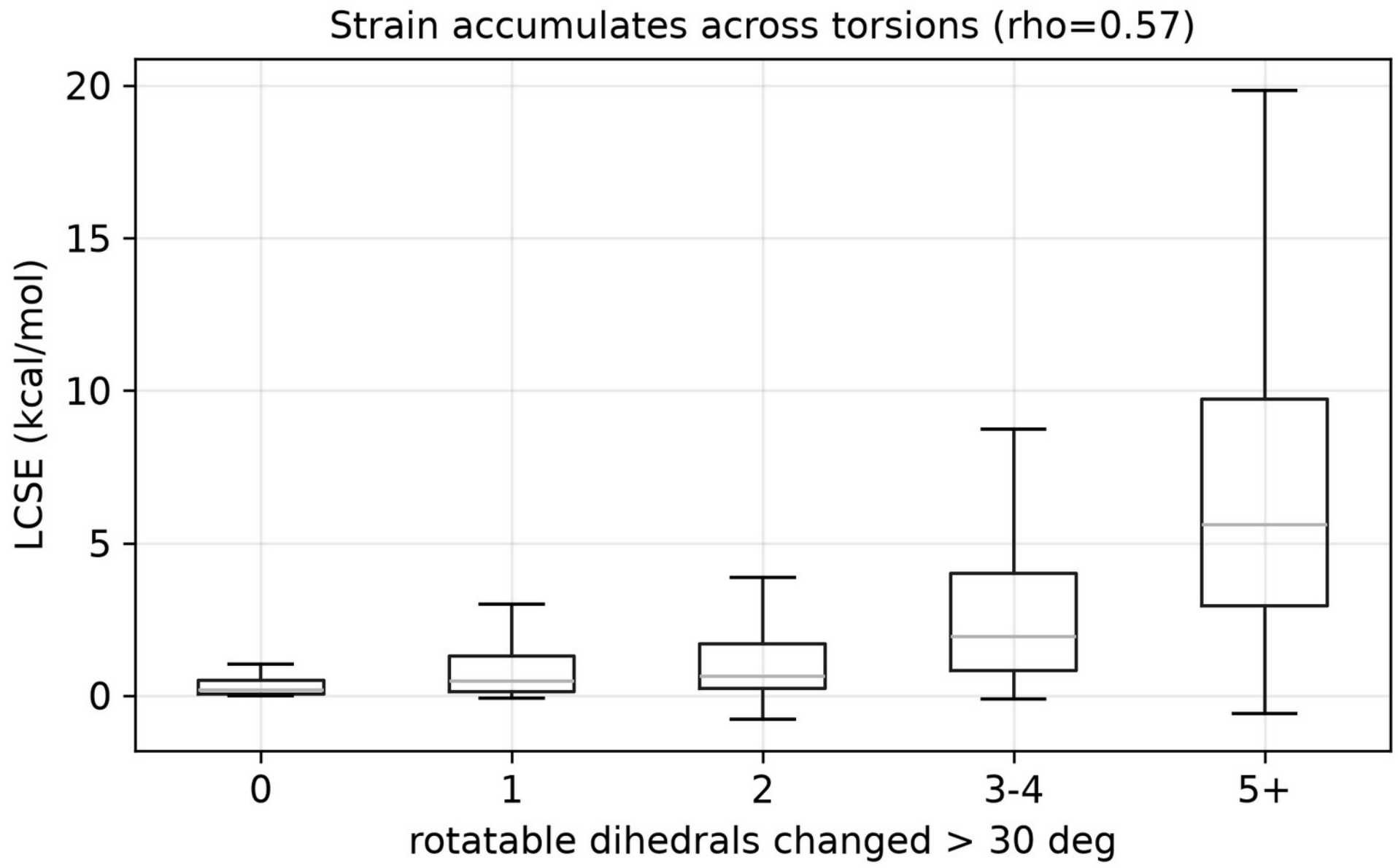

Figure S5. Ligand strain accumulates across many small torsional changes. LCSE for all 7887 ligands, binned by the number of rotatable-bond dihedrals that change by more than 30 degrees between the bound and global conformers (Spearman 0.57). The median rises from 0.2 kcal/mol when no torsion changes to 5.6 kcal/mol when five or more change, whereas the correlation with the single largest dihedral change is weak (Spearman 0.11), indicating that strain is distributed over many small adjustments rather than one large rotation. Boxes span the interquartile range, whiskers the 5th-95th percentiles.

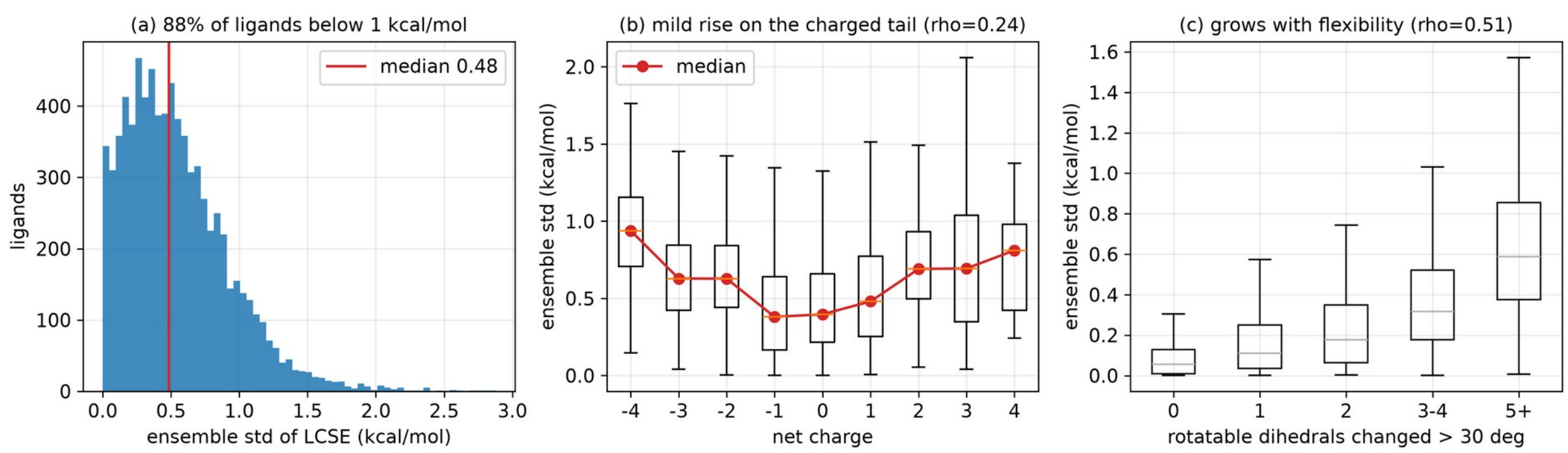

Figure S6. Ensemble uncertainty of the solvated ligand conformational strain energy (LCSE). For each of the 7,887 ligands the uncertainty is the standard deviation of the strain across the four AIMNet2-CPCM ensemble members, each member evaluated on both the bound and global conformers. (a) Distribution of the per-ligand ensemble standard deviation; the median is 0.48 kcal/mol and 88% of ligands fall below 1 kcal/mol. (b) Ensemble standard deviation by net charge; the spread rises mildly on the multiply charged tail, from about 0.4 kcal/mol for neutral and singly charged ligands to about 0.9 kcal/mol at charge -4. (c) Ensemble standard deviation versus the number of rotatable dihedrals that change by more than 30 degrees between the bound and global conformers; the uncertainty grows most strongly with conformational flexibility. This spread quantifies the internal consistency of the ensemble, not the accuracy of the underlying B97-3c/CPCM description.

2. LCSE distribution with respect to molecular properties

Table S1. The details of the ligands with LCSE>25 kcal/mol

Ligand ID	PDB ID	Chain	Residue number	Net Charge	LCSE
8DP	5MLD	G	301	-2	28.4
IR8	5VJN	B	201	-3	27.1
IR9	5VJP	A	201	-3	25.4
TSL	6J2B	A	301	-2	26.3
2SA	5NX9	C	501	-4	27.2
ATP	3TA0	D	200	-4	27.4
MHA	1IX1	B	1401	-1	31.4
APC	2YYE	A	501	-4	26.2
ATP	2XZW	D	200	-4	27.4
ATP	2YXU	B	2410	-4	28.3
ATP	3G59	A	305	-4	27.1
ATP	3I7V	A	135	-4	25.1
ATP	3IKH	C	900	-4	26.1
ATP	4MB2	B	301	-4	29.2
ATP	6GU5	A	303	-4	29.5
GNP	4JYB	A	401	-4	25.9
GTP	1RA7	A	1001	-4	25.0

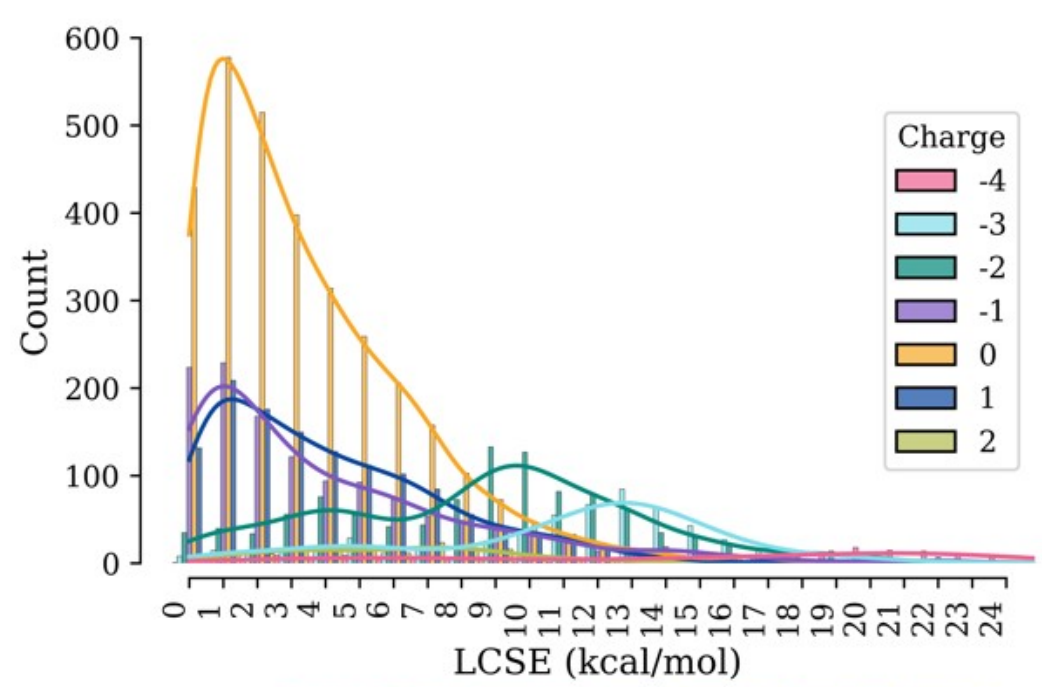

	-4	-3	-2	-1	0	1	2
Median	16.8	12.2	9.1	2.6	2.7	3.4	6.3
Mean	14.8	11.1	8.6	4.1	3.5	4.2	6.1
P75	21.0	14.0	11.5	6.0	5.1	6.2	8.4
P95	23.6	17.6	15.9	13.1	9.4	10.8	12.1

(a)

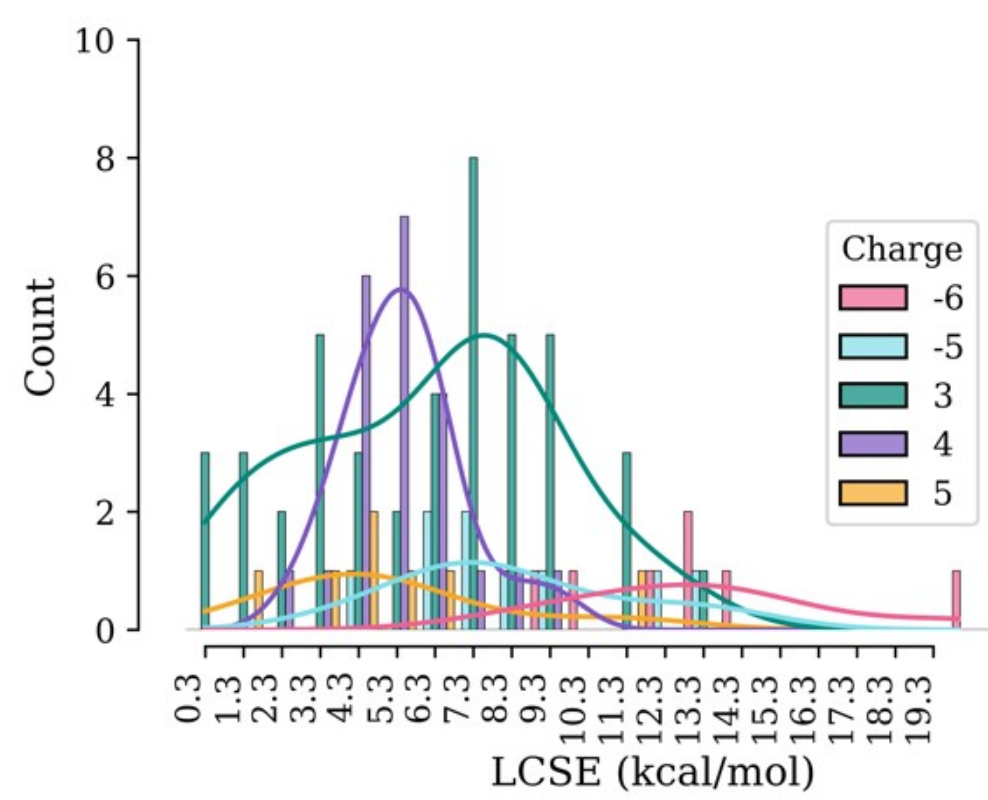

(b)

Figure S7. The distribution of the conformational ligand strain energies computed with respect to total charge. (a) The charge groups with large number of entries. (b) The charge groups with small number of entries.

2.1. Molecules that are not cofactors

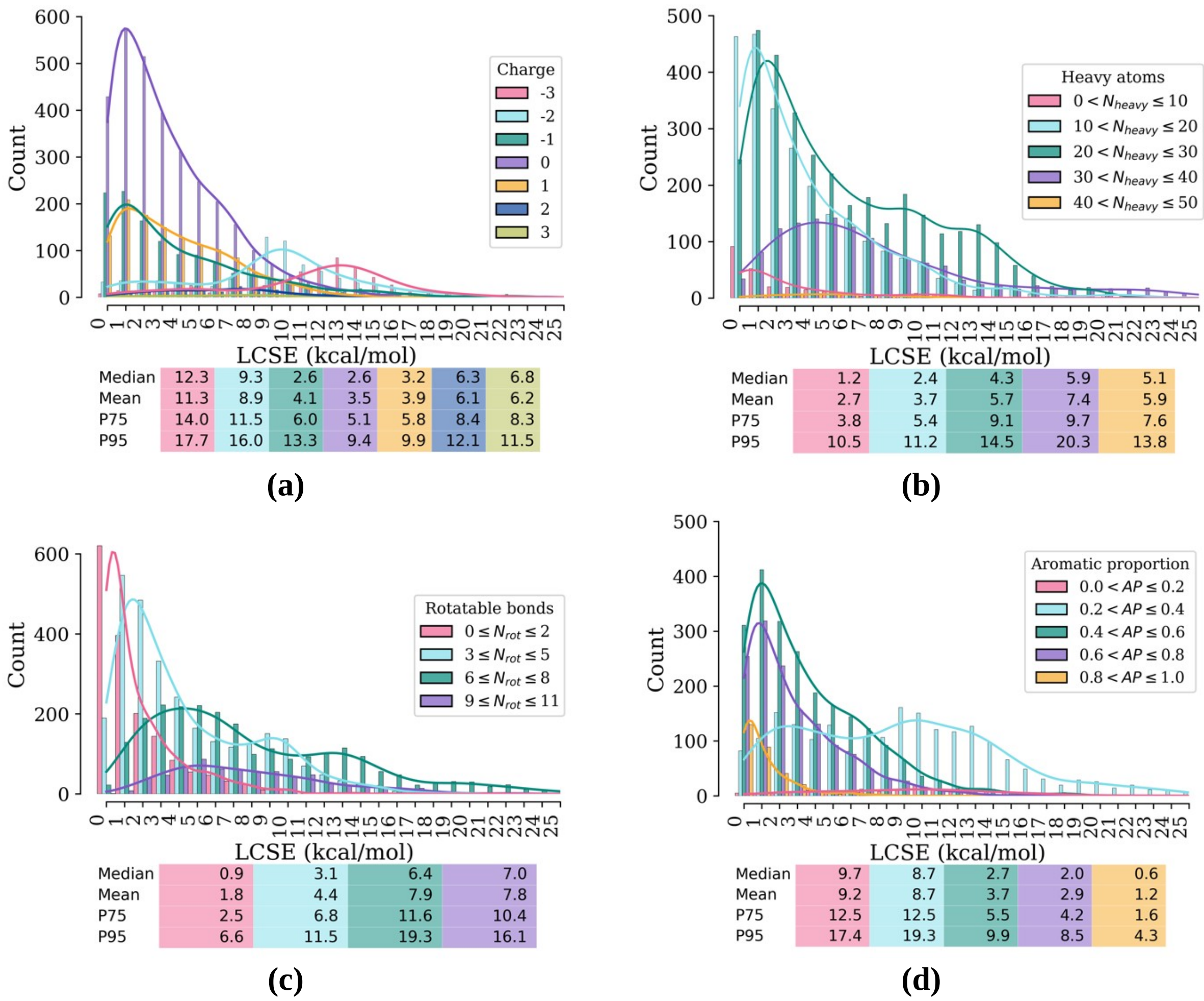

Figure S8. Conformational ligand strain energy profiles of ligands which are not cofactors. Strain energy distribution with respect to **(a)** total charge, **(b)** aromatic proportion, **(c)** number of heavy atoms, and **(d)** number of rotatable bonds as obtained with omega toolkit.

2.2. Molecules that bind to only one target

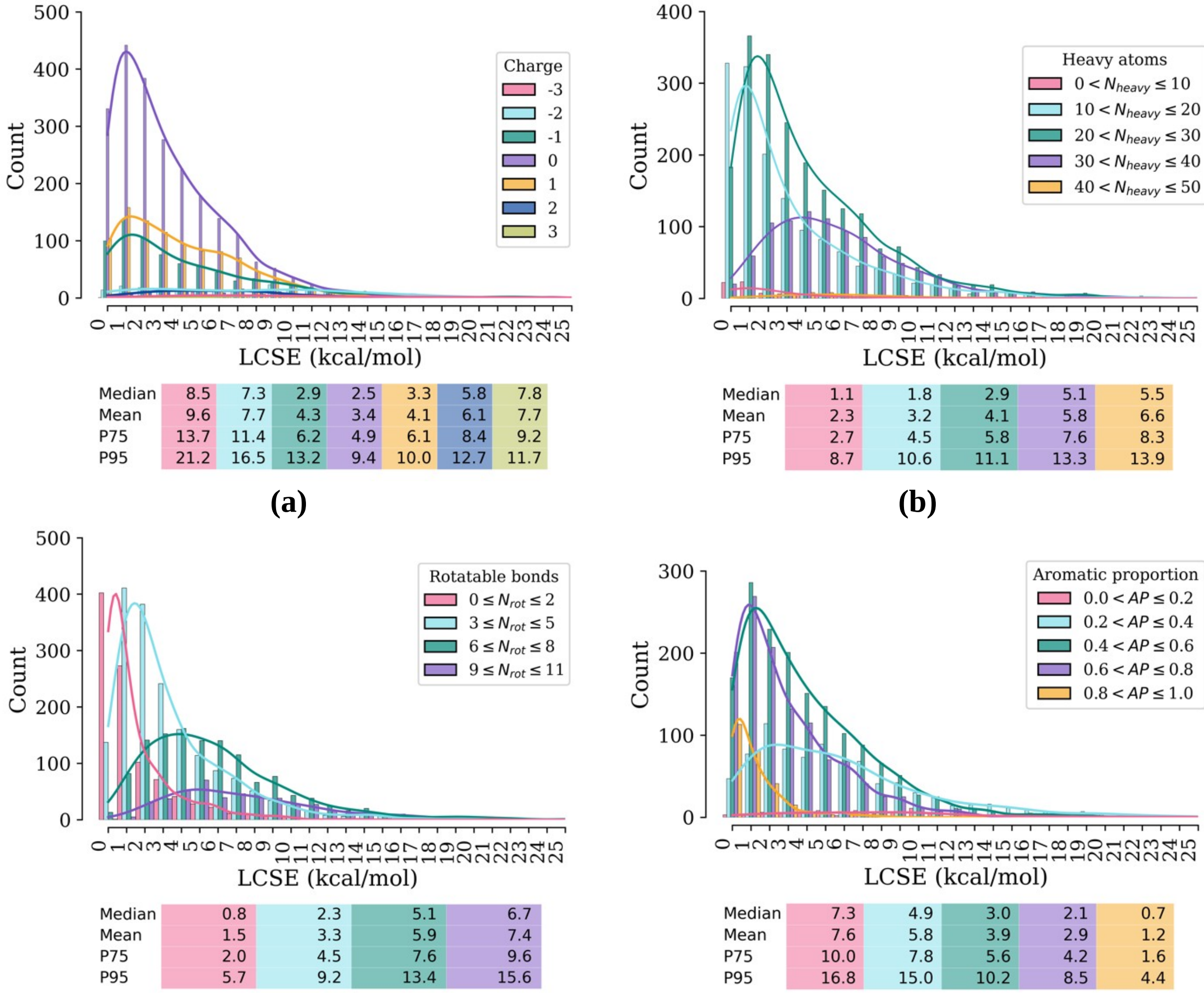

Median	8.5	7.3	2.9	2.5	3.3	5.8	7.8
Mean	9.6	7.7	4.3	3.4	4.1	6.1	7.7
P75	13.7	11.4	6.2	4.9	6.1	8.4	9.2
P95	21.2	16.5	13.2	9.4	10.0	12.7	11.7

(a)

Median	1.1	1.8	2.9	5.1	5.5
Mean	2.3	3.2	4.1	5.8	6.6
P75	2.7	4.5	5.8	7.6	8.3
P95	8.7	10.6	11.1	13.3	13.9

(b)

Median	0.8	2.3	5.1	6.7
Mean	1.5	3.3	5.9	7.4
P75	2.0	4.5	7.6	9.6
P95	5.7	9.2	13.4	15.6

(c)

Median	7.3	4.9	3.0	2.1	0.7
Mean	7.6	5.8	3.9	2.9	1.2
P75	10.0	7.8	5.6	4.2	1.6
P95	16.8	15.0	10.2	8.5	4.4

(d)

Figure S9 Conformational ligand strain energy profiles of ligands identified to bind only one target. Strain energy distribution with respect to **(a)** total charge, **(b)** aromatic proportion, **(c)** number of heavy atoms, and **(d)** number of rotatable bonds as obtained with omega toolkit.

2.3. Molecules that are classified as cofactors

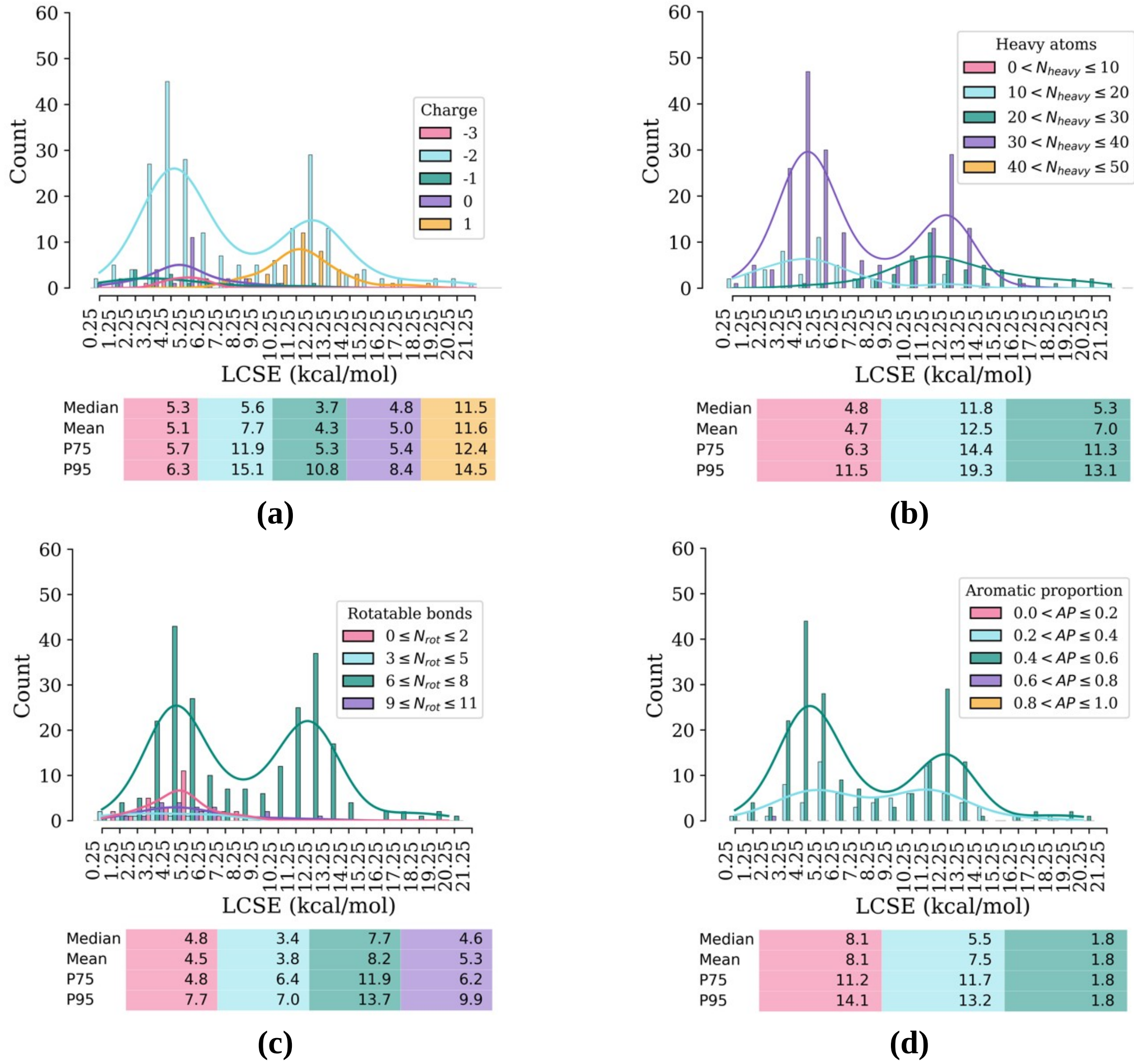

Figure S10. Conformational ligand strain energy profiles of ligands identified as cofactors. Strain energy distribution with respect to **(a)** total charge, **(b)** aromatic proportion, **(c)** number of heavy atoms, and **(d)** number of rotatable bonds as obtained with omega toolkit.

3. LCSE distribution of molecules that bind to multiple targets

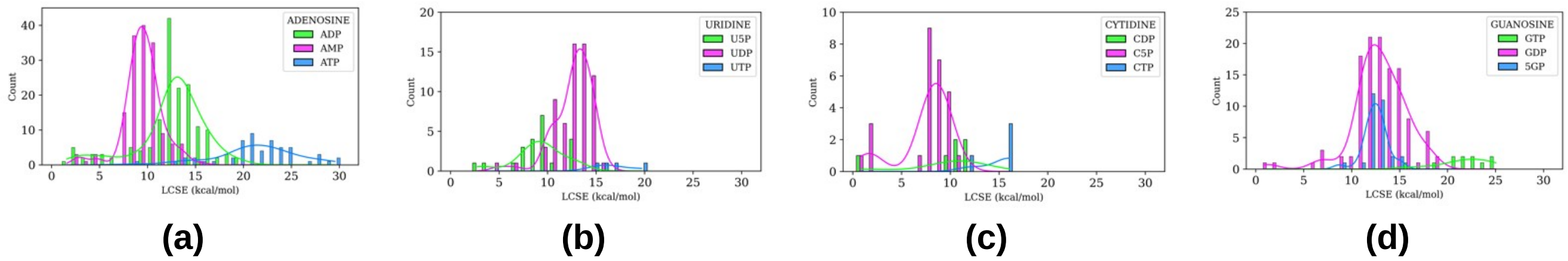

Figure S11. Conformational ligand strain energy profiles of ligands with phosphate groups. **(a)** Adenosine mono/di/tri-phosphate (**AMP**/**ADP**/**ATP**), **(b)** uridine-5'-mono/di/tri-phosphate (**U5P**/**UDP/UTP**), **(c)** cytidine-5'-mono/di/tri-phosphate (**C5P/CDP/CTP**), and **(c)** guanosine mono/di/tri-phosphate (**5GP/GDP**).

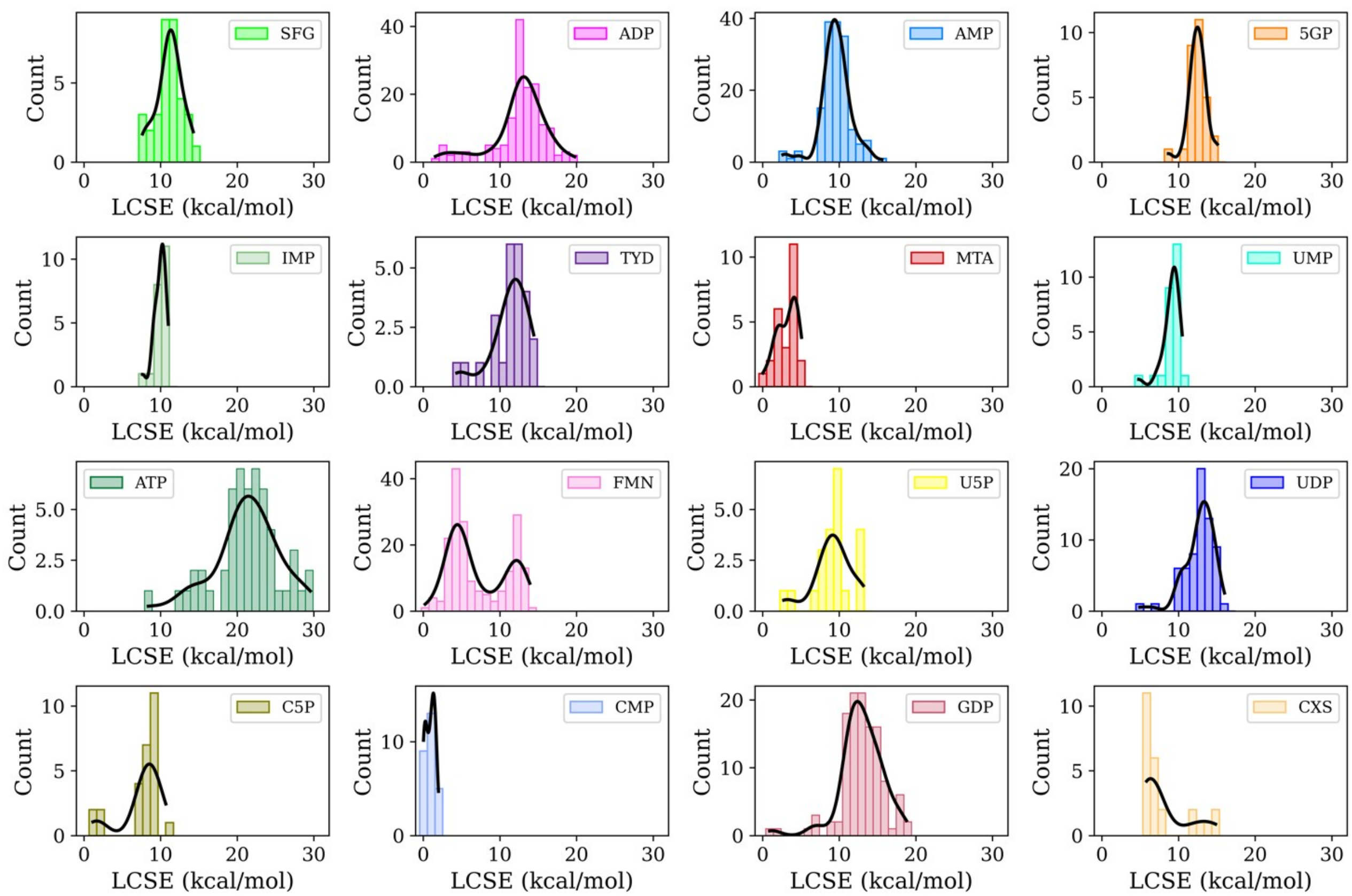

Figure S12. Conformational ligand strain energy profiles of ligands which bind to multiple targets. Ligands with less than 20 entries are not show. Sinefungin (**SFG**), adenosine mono/di/tri-phosphate (**AMP**/**ADP**/**ATP**), guanosine mono/di-phosphate (**5GP**/**GDP**), inosinic acid (**IMP**), thymidine-5'-diphosphate (**TYD**), 5'-deoxy-5'-methylthioadenosine (**MTA**), 2'-deoxyuridine 5'-monophosphate (**UMP**), flavin mononucleotide (**FMN**), uridine-5'-mono/di-phosphate (**U5P**/**UDP**), cytidine-5'-monophosphate (**C5P**), adenosine-3',5'-cyclic-monophosphate (**CMP**), 3-cyclohexyl-1-propylsulfonic acid (**CXS**).

4. LCSE distribution with respect to the enzyme subclasses

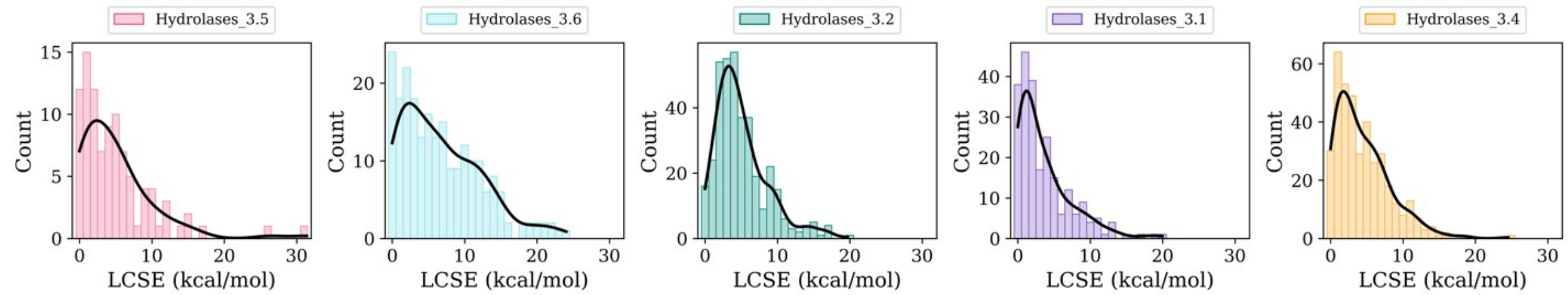

(a)

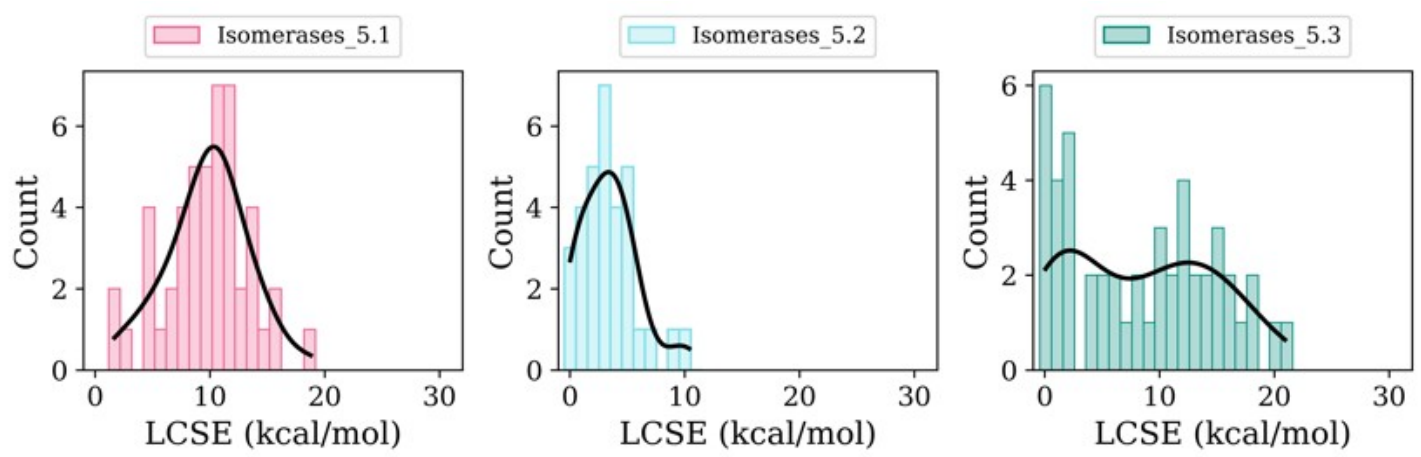

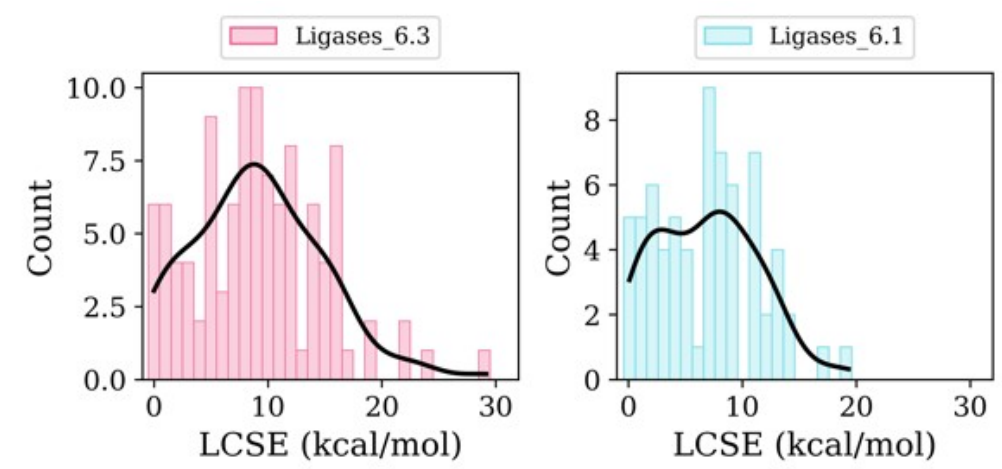

(b) **(c)**

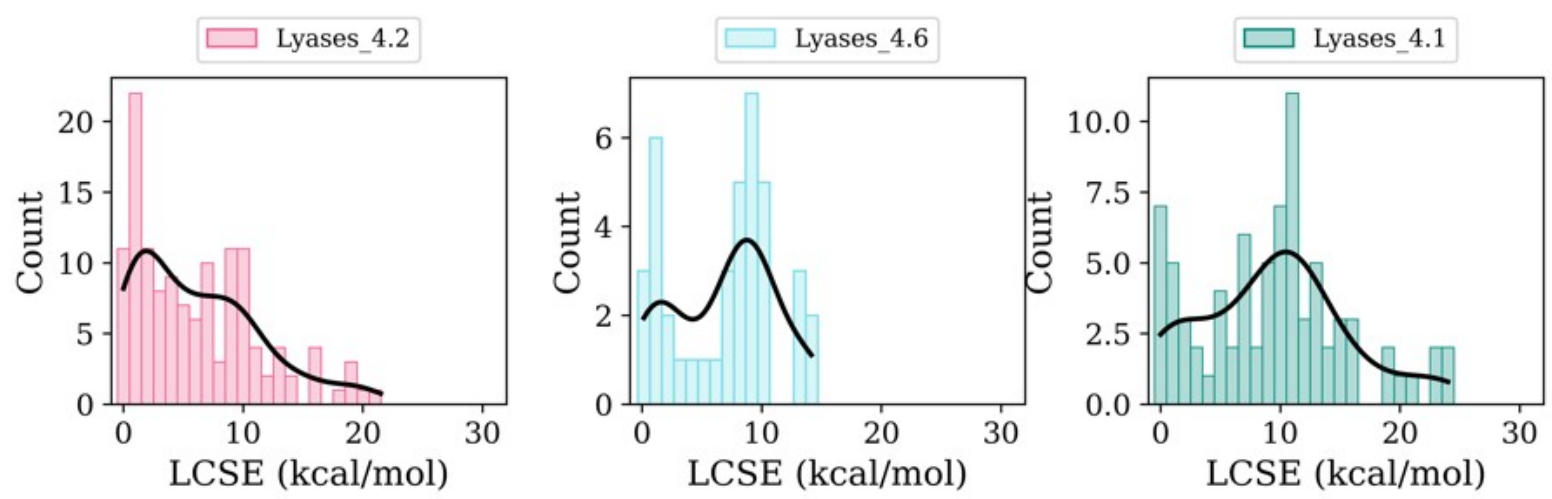

(d)

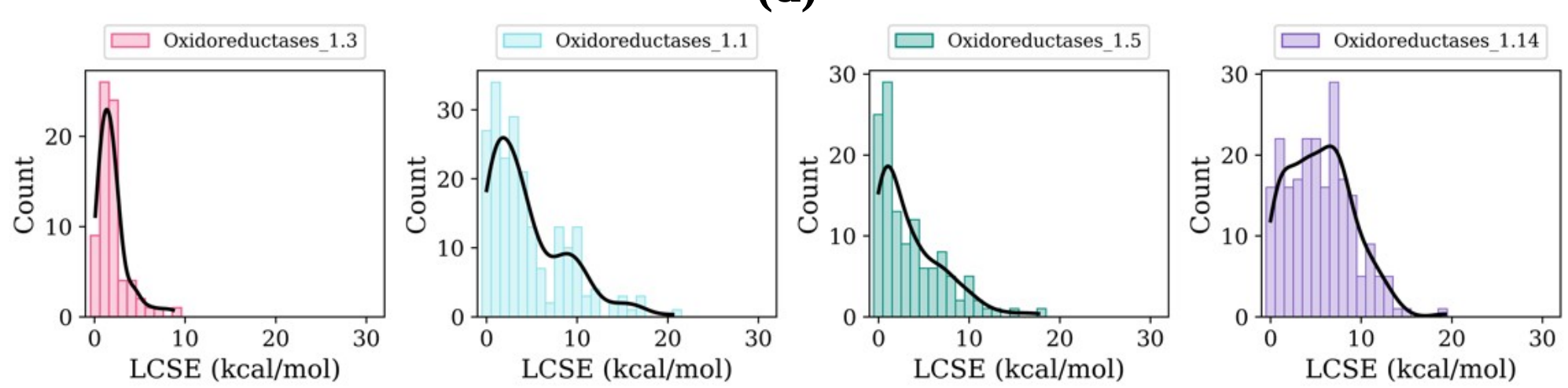

(e)

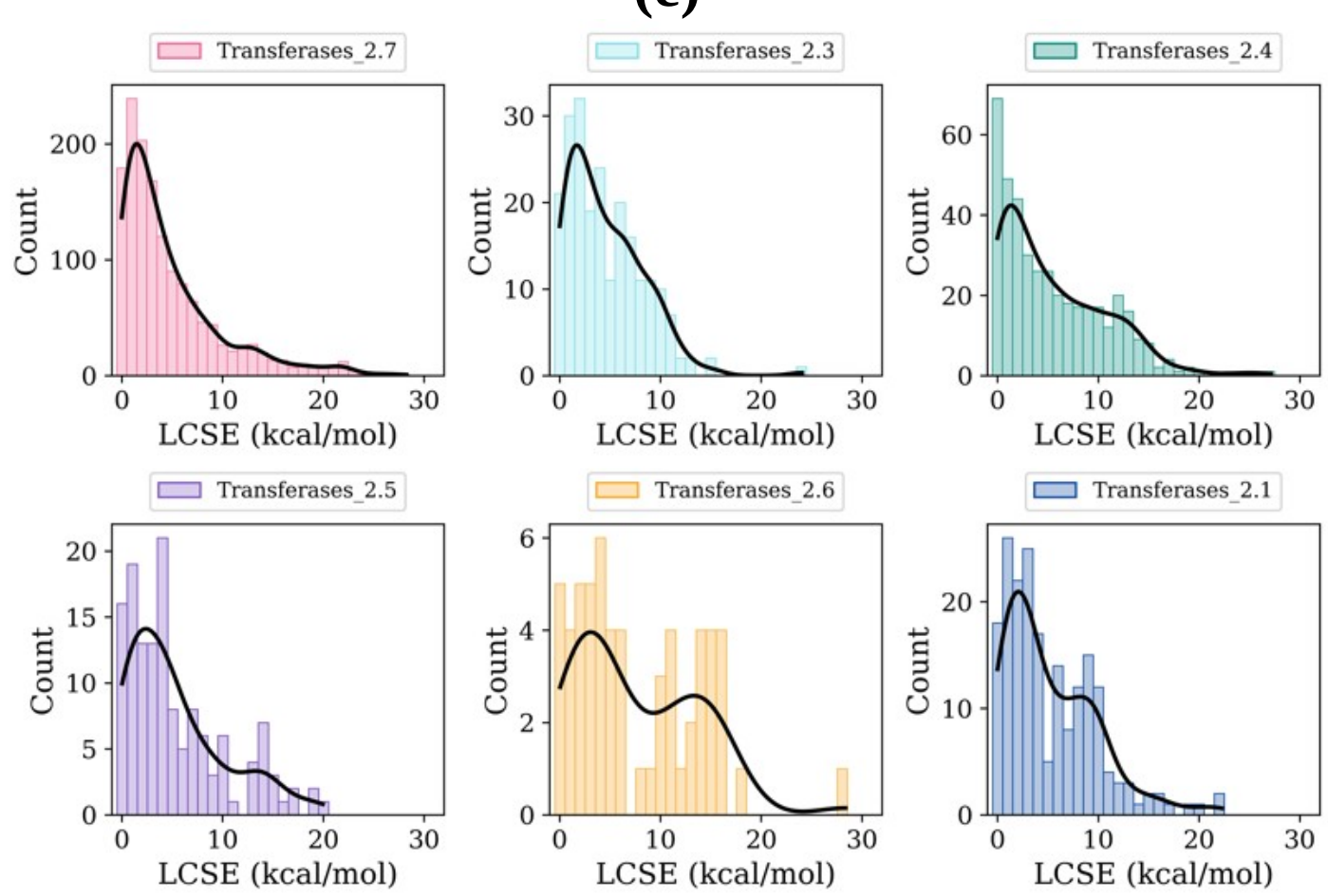

(f)

Figure S13. LCSE profile for ligands that are not identified as cofactors with respect to enzyme subclasses. Only enzyme classes with more than 20 entries are plotted. **(a)** Hydrolases, **(b)** Isomerases, **(c)** Ligases, **(d)** Lyases, **(e)** Oxidoreductases, and **(f)** Transferases.

Table S2. Identified enzyme classes within the dataset and total number of entries for them. Number of entries which are identified as cofactor are denoted within parenthesis.

**Enzyme Class**	**Number of Ligands**
Transferases	2551 (64)
Oxidoreductases	836 (95)
Isomerases	185 (2)
Hydrolases	1335 (3)
Ligases	218 (5)
Lyases	280 (3)
Translocases	16 (2)

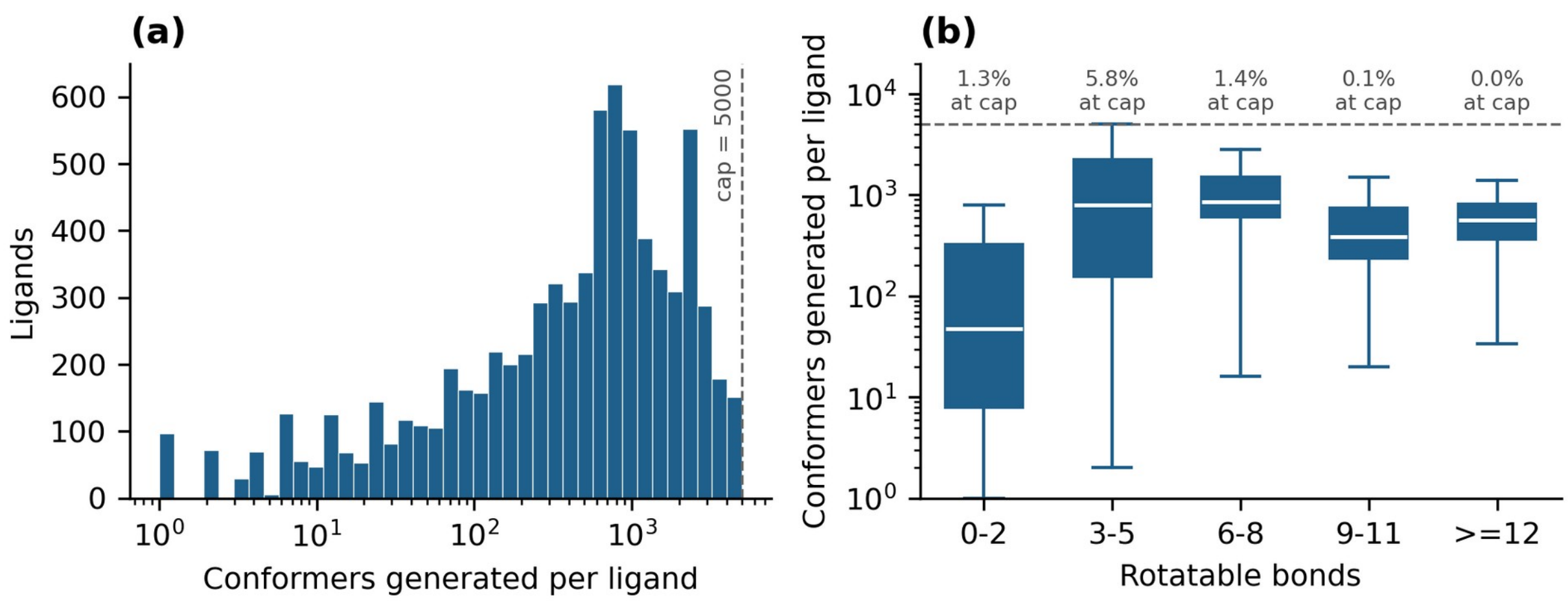

Figure S14. Conformer generation statistics. (a) Number of Omega conformers generated per ligand for the 7887 ligands, on a logarithmic scale. The dashed line marks the 5000-conformer cap, reached by 223 ligands (2.8%). (b) Conformers per ligand by number of rotatable bonds. Boxes span the interquartile range and whiskers extend to 1.5 IQR; the percentage of ligands at the cap is given above each box. The RMSD de-duplication threshold rises with rotor count, so the cap is reached mostly by ligands with 3 to 5 rotatable bonds.

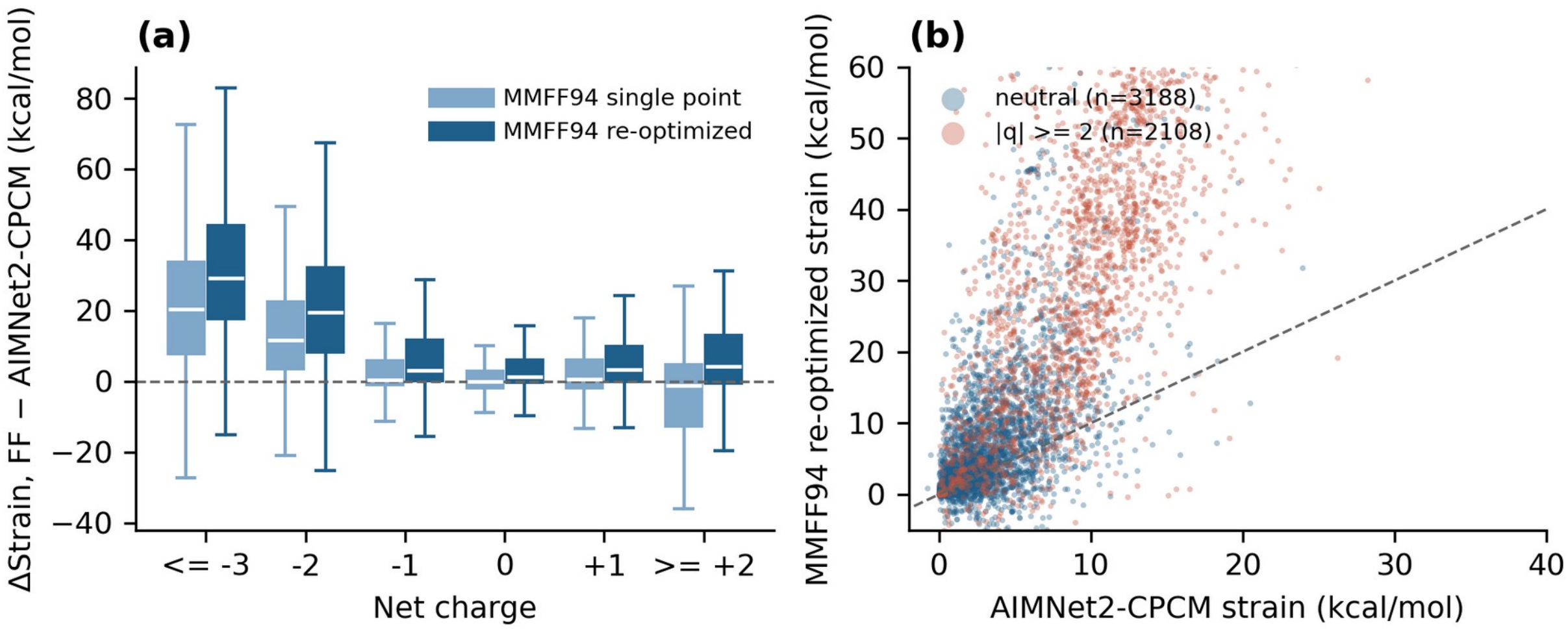

Figure S15. Force-field strain relative to AIMNet2-CPCM for all 7887 ligands. (a) Difference between MMFF94 strain and AIMNet2-CPCM strain by net charge. Light boxes: MMFF94 single points on the AIMNet2-CPCM conformers. Dark boxes: both endpoints re-optimized on the MMFF94 surface, with the rotatable-bond dihedrals of the bound conformer restrained to their deposited values and the global conformer unrestrained. Boxes span the interquartile range; whiskers extend to 1.5 IQR. (b) Re-optimized MMFF94 strain against AIMNet2-CPCM strain for neutral ligands and for ligands with |q| ≥ 2; the dashed line is the identity. Summary statistics are given in Table S4.

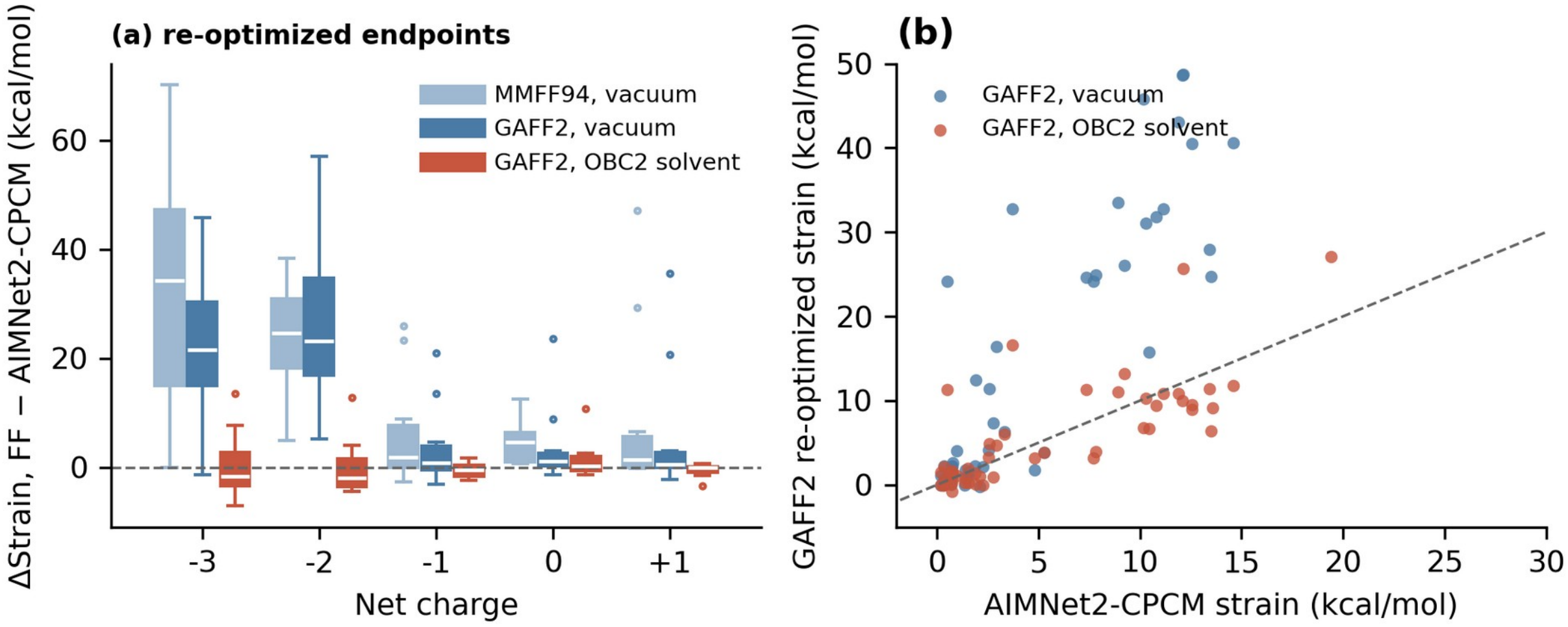

Figure S16. Effect of implicit solvation on force-field strain for a stratified subset of 50 ligands, 10 per net charge class (0, +1, −1, −2, −3), with 12 to 40 heavy atoms. (a) Difference between re-optimized force-field strain and AIMNet2-CPCM strain for MMFF94 in vacuum, GAFF2 with AM1-BCC charges in vacuum, and GAFF2 in the OBC2 generalized Born model. GAFF2 calculations used OpenMM 8 with openmmforcefields and gaff-2.11. (b) Re-optimized GAFF2 strain in vacuum and in OBC2 against AIMNet2-CPCM strain; the dashed line is the identity. Summary statistics are given in Table S5.

Table S3. Cost of one geometry optimization per method and extrapolation to the 8.16 million conformers of this work. All measurements from this work were made on one node with an AMD EPYC 7H12 CPU and an NVIDIA L40S 48 GB GPU, using the single ensemble member used in production. The production runs themselves ran on NVIDIA V100 GPUs and were not timed at the conformer level. Unbatched rows: 32 ligands stratified by size, 8 each with fewer than 20, 20 to 30, 30 to 40 and 40 or more heavy atoms (18 to 80 atoms in total, net charge −4 to +2), one RDKit ETKDG conformer each, ASE FIRE to fmax < 0.05 eV/Å, single-threaded on the CPU. Times are the wall-clock of a second, warm run after a discarded first run. One GFN2-xTB optimization did not converge and is included at its wall time. Batched rows: 1024 ETKDG conformers of 64 ligands with a mean of 51 atoms, SaddleForge L-BFGS with NORMAL force convergence on fmax and frms, at most 500 steps, second of two runs. Throughput was 21.0 conformers per s at batch 256 (11.8 GiB) and 20.1 at batch 1024 (35.8 GiB, first run), so the GPU is saturated from batch 256 on for ligands of this size. The unbatched AIMNet2-CPCM rows use the full four-member ensemble on the CPU and on the GPU; the single-member CPU time is 3.0 s. One conformer at a time gains only a factor of 3 from the GPU, because each step is one kernel launch sequence regardless of batch size. Forward-pass throughput is the mean of 10 evaluations after 3 warm-up evaluations at batch 256, padded to 56 atoms. Extrapolations multiply the per-conformer value by 8 164 128 and are order-of-magnitude estimates. The CPU rows are reference points only. The AIMNet2 PyTorch implementation is not optimized for CPU execution, and unbatched GPU execution is launch-bound, so the cost of the method is the batched GPU row. The CREST estimate combines the 400× ratio and the 44 s per ligand reported for StrainRelief and is indicative only.

Method	Hardware	Per-conformer optimization	Extrapolated, 8.16 M conformers	Per ligand (mean 1035 conformers)	Source
MMFF94 (RDKit 2025.09)	1 CPU core	0.05 s (median)	~110 CPU-h	~0.9 min	this work, 32 conformers
GFN2-xTB / ALPB(water), xtb 6.7	1 CPU core	7.7 s (median)	~17 000 CPU-h	~2.2 h	this work, 32 conformers
AIMNet2-CPCM, one ensemble member, unbatched, ASE FIRE (PyTorch on CPU, not the intended platform)	1 CPU core, 1 thread	3.0 s (median; 112 steps, 0.023 s per step)	~6800 CPU-h	~52 min	this work, 32 conformers
AIMNet2-CPCM, four-member ensemble, unbatched, ASE FIRE	1 CPU core, 1 thread	11.6 s (median; 107 steps, 0.084 s per step)	~26 000 CPU-h	~3.3 h	this work, 32 conformers
AIMNet2-CPCM, four-member ensemble, unbatched (batch 1), ASE FIRE	NVIDIA L40S	3.7 s (median; 0.035 s per step; launch-bound)	~8400 GPU-h	~64 min	this work, 32 conformers
AIMNet2-CPCM, one member, forward pass only, batch 256	NVIDIA L40S	10 500 conformer-steps per s (0.024 s per batch step; ~240× one CPU core)			this work; batch-size scan in Table S6
AIMNet2-CPCM, one member, batched L-BFGS (SaddleForge), batch 512	NVIDIA L40S	22.7 conformers per s (0.044 s per conformer; median 161 steps; 1022/1024 converged; 18.5 GiB peak memory)	~100 GPU-h	~46 s	this work, 1024 conformers
Production runs (one member, in-house batched optimizer)	NVIDIA V100	not timed at conformer level			this work
MACE (StrainRelief), ASE BFGS, 20 conformers	GPU (unspecified)	~2.2 s per conformer (44 s per ligand for 20 conformers)	n/a	44 s (20 conformers); 184 s (100)	ref 16
GFN2-xTB + CREST (iMTD-GC), 50	CPU	~400× slower than the	~4 × 10^4 CPU-	~5 CPU-h	ref 16

conformers		MACE protocol	h (estimate)	(estimate)	
B97-3c/CPCM (ORCA 5.0.3), single point	CPU	minutes per conformer	not feasible at this scale		this work

Table S4. MMFF94 strain versus AIMNet2-CPCM strain by net charge class for all 7887 ligands (one ligand failed MMFF94 typing). SP: single point on AIMNet2-CPCM conformers; re-opt: both endpoints re-optimized on the MMFF94 surface. Bias is the median of MMFF94 minus AIMNet2-CPCM strain, and MAD is the median absolute difference; all energies are in kcal/mol.

Net charge	n	Median LCSE, AIMNet2-CPCM	Median, MMFF94 SP	Median, MMFF94 re-opt	Median bias SP	Median bias re-opt	MAD SP	MAD re-opt	Spearman SP	Spearman re-opt
≤ −3	800	12.6	33.2	42.4	20.3	29.2	20.6	29.2	0.77	0.75
−2	1070	9.1	19.9	28.7	11.6	19.6	12.4	19.7	0.73	0.69
−1	1291	2.6	2.7	6.0	0.4	3.1	2.9	3.9	0.67	0.78
0	3188	2.7	2.1	4.2	0.0	1.3	2.2	2.5	0.56	0.62
+1	1299	3.4	3.9	7.0	0.7	3.3	3.5	4.1	0.61	0.64
≥ +2	238	6.2	2.2	9.6	-1.1	4.2	8.4	6.3	0.24	0.37

Table S5. Force-field strain minus AIMNet2-CPCM strain on the stratified 50-ligand subset of Figure S16: median bias with the median absolute difference in parentheses, in kcal/mol. SP: single point on the AIMNet2-CPCM conformers. Re-opt: both endpoints re-optimized on the respective force-field surface, with the rotatable-bond dihedrals of the bound conformer restrained to their deposited values.

Net charge	n	MMFF94 re-opt	GAFF2 vac SP	GAFF2 vac re-opt	GAFF2/OBC2 SP	GAFF2/OBC2 re-opt
−3	10	34.2 (34.2)	21.9 (21.9)	21.6 (21.6)	2.0 (2.2)	-1.7 (3.8)
−2	10	24.6 (24.6)	20.9 (20.9)	23.1 (23.1)	-2.9 (3.6)	-1.9 (3.4)
−1	10	1.8 (2.4)	-0.4 (2.8)	0.9 (1.9)	-0.8 (0.8)	-0.5 (1.5)
0	10	4.6 (4.6)	-0.4 (1.2)	1.2 (1.4)	-0.7 (1.0)	0.3 (1.2)
+1	10	1.4 (1.4)	-0.2 (1.1)	0.5 (1.1)	-0.7 (0.7)	-0.1 (0.5)

Table S6. Forward-pass (energy and forces) throughput of AIMNet2-CPCM on one NVIDIA L40S as a function of batch size, for the full four-member ensemble and for the single member used in the production conformer search. Batches are contiguous slices of the size-sorted pool of 2048 conformers of library ligands (64 ligands, 32 ETKDG conformers each, mean 51 atoms), padded to the largest molecule in the batch. Times are the mean of 10 evaluations after 3 warm-up evaluations. Conformer-steps per second = batch size / time per step. Throughput per conformer falls with batch size above 256 because larger batches pull in larger molecules and more padding, not because the GPU is short of work. The batched optimizer used for the cost estimate (Table S3) therefore runs at batch 256 to 512. The last two rows are per-step times of unbatched ASE FIRE optimizations of 32 ligands with 18 to 80 atoms, on the GPU and on one CPU thread of the AMD EPYC 7H12, for scale.

Batch size	Max atoms (padded)	Ensemble (4 members): s per step	Ensemble: conformer-steps per s	Ensemble: GPU memory (GiB)	One member: s per step	One member: conformer-steps per s	One member: GPU memory (GiB)
256	56	0.103	2490	1.8	0.024	10 550	1.8
512	59	0.240	2130	3.8	0.060	8550	3.8
1024	67	0.630	1620	9.4	0.157	6510	9.4
2048	93	2.11	970	33.7	0.527	3890	33.5
1, GPU (ASE)	18 to 80	0.035	29				
1, CPU thread (ASE)	18 to 80	0.084	12		0.023	44	
